# Low-Cost Temperature Sensing Reveals Thermal Signatures of Microbial Activity in Winogradsky Columns

**DOI:** 10.3390/s25237146

**Published:** 2025-11-22

**Authors:** Ahmad Itani, Dario Mager, Kersten S. Rabe, Christof M. Niemeyer

**Affiliations:** 1 Biomolecular Micro- and Nanostructures, Institute for Biological Interfaces 1 (IBG-1), Karlsruhe Institute of Technology (KIT), Hermann-von-Helmholtz-Platz 1, D-76344 Eggenstein-Leopoldshafen, Germany; on6879@partner.kit.edu (A.I.); kersten.rabe@kit.edu (K.S.R.); 2 Institute for Microstructure Technology (IMT), Karlsruhe Institute of Technology (KIT), Hermann-von-Helmholtz-Platz 1, D-76344 Eggenstein-Leopoldshafen, Germany; dario.mager@kit.edu

**Keywords:** microbiome analysis, thermogenesis, Winogradsky columns, model ecosystems, instrumentation, data analysis

## Abstract

Temperature is a key driver of microbial metabolism, yet non-invasive methods for quantifying microbially generated heat in complex environments remain limited. Here, we present a low-cost digital temperature sensing system integrated into an Arduino-controlled data acquisition setup to monitor microbial activity in stratified Winogradsky columns, which are self-contained sediment microcosms that reproduce natural oxygen and sulfide gradients. Localized temperature differences of up to 0.55 ± 0.04 °C were detected between aerobic and anaerobic layers, consistent with microbial heat generation in active sediment zones. Short-term insulation experiments further amplified these effects, demonstrating that microbial thermogenesis can serve as a reliable proxy for metabolic activity. Compared with infrared thermography or isothermal microcalorimetry, the proposed approach is simple, cost-effective, and compatible with aqueous and stratified systems. The method enables real-time, non-invasive observation of microbial metabolic dynamics and establishes a framework for continuous thermal monitoring in living environmental microcosms.

## 1. Introduction

Temperature is among the key physicochemical factors shaping microbial growth and community composition, alongside pH [1], CO_2_ and O_2_ availability [2], water activity [3], osmotic pressures [4], and light [5]. Microorganisms are classified by their temperature ranges into psychrotrophs, psychrophiles, mesophiles, and thermophiles [6], each with defined minimum, optimum, and maximum temperatures. This temperature dependence directly reflects underlying metabolic activity, since microbial cells continuously catabolize environmental resources, releasing heat as a by-product of biochemical reactions. Such microbial thermogenesis [7], observed macroscopically in compost where temperatures can exceed 60 °C [8], provides a means to probe metabolism through thermal signatures. The heat released by metabolic pathways follows Hess’s law, representing the enthalpy difference between products and substrates [9,10], and is largely conserved across aerobic oxidations, yielding a nearly constant heat per mole O_2_ known as Thornton’s constant [11]. Deviations from this constant can thus reveal shifts in the dominant metabolic pathways [12].

While this principle is well established, the direct measurement of microbial thermogenesis in natural or complex living systems remains challenging. Conventional calorimetric approaches such as isothermal microcalorimetry (IMC) [13,14] can detect metabolic heat from as few as 10^5^ cells, generating characteristic thermograms that mirror microbial growth dynamics [15,16]. IMC has enabled quantification of growth phases [15], rapid identification of pathogens [17], and analysis of soil metabolic profiles with depth gradients [18], but its closed-vial design imposes anoxic conditions, long equilibration times, and artificial constraints on natural gradients [13]. Other temperature-sensing strategies, including fluorescence thermometry [19,20], infrared imaging [20], and nanoscale pipette electric thermography (NET) [21], offer high spatial precision but are either invasive, costly, or incompatible with aqueous media where infrared radiation is absorbed. To date, none of these approaches has implemented direct temperature sensing within living, water-rich microbial ecosystems while resolving spatial thermal gradients in real time. This methodological gap underscores the need for sensing strategies that are compatible with aqueous environments and capable of continuous, spatially resolved temperature measurements within intact, structured microbial systems.

**Figure 1:**
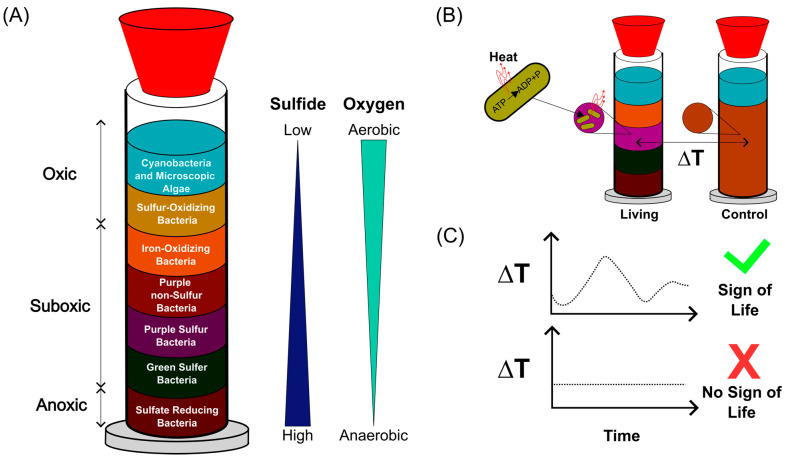
Concept of temperature-based monitoring of microbial activity in Winogradsky columns. (**A**) Schematic representation of a Winogradsky column showing the formation of metabolically distinct microbial layers along oxygen and sulfide gradients. Adapted from [26,28,29]. (**B**) Conceptual illustration of microbial thermogenesis: active microorganisms generate heat during metabolism, leading to measurable local temperature increases in living columns, whereas no such change is expected in sterilized controls. (**C**) Processing of temperature data using automated analysis scripts allows identification of thermal patterns characteristic of microbial activity, providing a non-invasive indicator of life within complex sediment systems.

Model ecosystems such as the Winogradsky column provide a controlled yet ecologically relevant platform for such studies [22,23,24]. First described by Sergei Winogradsky [25], these sediment-based microcosms reproduce oxygen and sulfate gradients that drive microbial self-organization into metabolically distinct layers [26,27,28]. Here, we investigate whether temperature could serve as a non-invasive, real-time proxy for microbial metabolic activity within such stratified systems (Figure 1).

While this study does not introduce new sensor hardware, it presents a novel application of established digital thermometers for detecting microbial heat production in situ. By embedding these low-cost sensors directly within a living Winogradsky column, we demonstrate for the first time that continuous temperature monitoring can reveal localized metabolic activity. This methodological approach extends the use of low-cost sensor platforms beyond educational and analytical contexts, offering a scalable tool for real-time environmental microbiology.

This study tests the hypothesis that microbial metabolic heat can be detected in complex, living sediment ecosystems through continuous temperature monitoring. By embedding low-cost digital temperature sensors within Winogradsky columns, we aimed to determine whether localized temperature differences could serve as indicators of microbial metabolic activity. This approach establishes temperature as a potential non-invasive proxy for microbial processes in stratified environments and provides a framework for developing accessible, real-time sensing systems for microbial ecology.

## 2. Materials and Methods

### 2.1. Sediment Collection and Post-Processing

Benthic sediment was collected on 23 December 2021 from a shallow freshwater pond in the Saalbachniederungen nature reserve near Hambrücken, Germany (49.1557 N, 8.5469 E). The material was passed through a 6 mm soil sieve to remove coarse particles and debris. To determine the water content and estimate the sediment-specific heat capacity, nine subsamples were placed in pre-weighed aluminum dishes and dried in a B5042 incubator (Heraeus Group, Hanau, Hessen, Germany) at 70 °C for 24 h. The mass of each sample was recorded at defined intervals using a Kern EMB-V precision balance (Kern GmbH Balingen-Frommern, Balingen, Baden-Württemberg, Germany) until no further weight change was detected, indicating complete evaporation of water. The respective values are summarized in Table S1 in the Supporting Information. The average dry-to-fresh mass ratio was 38.2%, consistent with previous measurements [30], and this value was subsequently used to approximate the thermal properties of the sediment. Unused sediment was aliquoted into glass bottles and stored at −80 °C until used.

### 2.2. Assembly of Winogradsky Columns and Sensor Integration

Winogradsky columns were prepared using transparent polymethyl methacrylate (PMMA, sourced from in-house stock in KIT) cylinders (inner diameter 4 cm, height 20 cm). Each cylinder was sealed at the bottom with a hexagonal PMMA plate using a hot-melt adhesive and closed at the top with an air-tight rubber stopper (MOCAP, Zerniki, Poland) (Figure S1). The total column volume was 200 mL. To promote microbial stratification in the living column, a sulfide gradient was pre-established following a modified protocol by Esteban et al. [27]. Sediment was divided into two portions: (i) an enriched slurry containing 4.5 g dried leaf litter, 5 g CaSO_4_, and 5 g CaCO_3_ per 100 mL of wet sediment, and (ii) an unenriched slurry composed of native sediment only. The column was first filled with 50 mL of enriched sediment (≈4 cm height), overlaid with 150 mL of unenriched sediment (≈16 cm height), and sealed with the rubber stopper. For the abiotic control, 200 mL of the original sediment was autoclaved (VARIOKLAV 135 S, HP Medizintechnik, Oberschleißheim, Bayern, Germany) for 20 min at 121 °C prior to column assembly.

**Figure 2:**
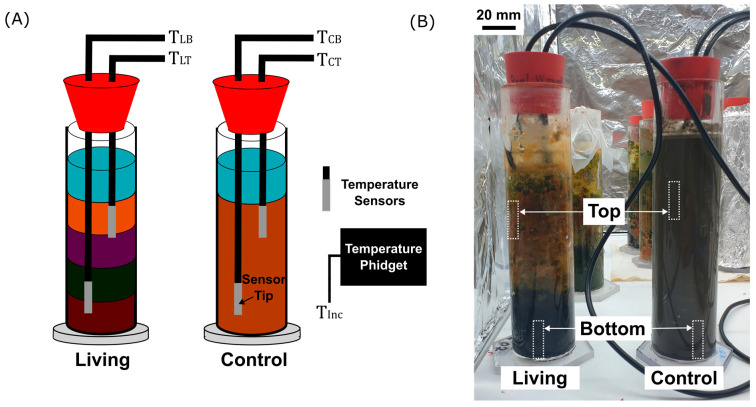
Temperature sensor configuration and experimental setup. (**A**) Schematic representation of the Winogradsky columns showing the estimated positions of waterproof DS18B20 temperature sensors embedded in the top and bottom sediment layers of both the living and control columns. This enabled the monitoring of the temperature in the top and bottom regions of the living and control columns (Abbreviations: T_LT_ = living top, T_LB_ = living bottom, T_CT_ = control top, T_CB_ = control bottom). An additional Temperature Phidget sensor monitored the ambient incubator temperature (T_Inc_). (**B**) Photograph of the experimental setup with the living column (**left**) and the sterilized control column (**right**). Dotted boxes indicate the approximate locations of the temperature sensors within the columns. Scale bar is 20 mm.

To investigate whether temperature dynamics can serve as an indicator of microbial activity, a low-cost temperature sensing system was implemented in the living and abiotic control Winogradsky columns (Figure 2A). Two waterproof digital thermometers (DS18B20 sensors, Analog Devices Inc., Norwood, MS, USA, waterproofed version from DFRobot, Shanghai, China) were integrated through holes drilled into the rubber stopper using a bench drill (PBD 40, Bosch, Gerlingen, Baden-Württemberg Germany). The sensors were positioned at two depths: the top region (~8 cm below the column neck) and the bottom region (~18 cm depth). This arrangement ensured that both sensors were fully embedded within the sediment matrix, with the upper sensor located just beneath the water–sediment interface and the lower sensor positioned at the base of the column. The stainless-steel sensor probes were inserted through holes drilled into the rubber stoppers sealing each column and secured in place (Figure 2B, dotted boxes “Top” and “Bottom”). The resulting temperature readings correspond to T_LT_ (living top), T_LB_ (living bottom), T_CT_ (control top), and T_CB_ (control bottom). An additional sensor (Temperature Phidget, Phidgets Inc., Calgary, AB, Canada) monitored the ambient incubator temperature (T_Inc_) to correct for environmental fluctuations. All sensors were connected to the Arduino microcontroller and logged continuously via Python (v3.13.2), which recorded temperature values together with their corresponding date and time in structured Excel files.

### 2.3. Incubator

The complete setup was placed in the controlled-illumination incubator (90 × 72 × 55 cm, Figure S2) equipped with three SOLAR STINGER^®^ LED lamps (25 W each, 72 cm length from ECONLUX, Cologne, North Rhein-Westphalia, Germany) mounted 25 cm apart to provide uniform light distribution. The chamber remained open at the top to allow visual inspection of the columns. Inner walls were lined with aluminum foil to enhance thermal insulation and light reflection. Illumination was regulated using the ECONLUX con1 control unit (setting: SOLAR STINGER^®^ SunStrip III 35 FRESH). According to the manufacturer’s specifications [31], each lamp provides a lighting intensity of 43.91 µmol s^−1^. Since three identical lamps were used, the total photon flux amounted to 131.7 µmol s^−1^. With the incubator footprint of 72 × 55 cm (0.396 m^2^), the resulting average light intensity was 333 µmol m^−2^ s^−1^. The incubator temperature, monitored with a Temperature Phidget sensor (Phidget Inc., Calgary, AB, Canada), fluctuated between 22 °C and 30 °C during the experiments. Perturbations caused by external factors, such as periodic switching of laboratory lights, are addressed in Section 3.3. A 24-h day–night cycle was programmed as follows: light intensity increased gradually from 0% at 07:00 to 100% (25 W) by 08:00, remained constant until 00:00, and then dimmed to 1.9% blue light by 01:00 to simulate moonlight. This 18-h light/6-h dark schedule ensured that the dark phase occurred entirely during nighttime, thereby avoiding interference from natural daylight.

### 2.4. Sensor and Circuit

Temperature measurements were performed using digital DS18B20 thermometers (Analog Devices, formerly Maxim Integrated, Norwood, MS, USA), which record temperature in °C with 9–12-bit resolution. At the default 12-bit setting, the device provides a resolution of 0.0625 °C and an accuracy of ±0.5 °C. Communication with the microcontroller was achieved via the 1-Wire^®^ bus protocol [32], which requires a single data line and ground connection. Each sensor includes three leads corresponding to power (VDD), ground (GND), and data output (DQ) (Figure S3). For this study, waterproof encapsulated DS18B20 probes were employed, allowing stable operation within the aqueous sediment environment of the Winogradsky columns. Electrical connections between the sensors and the Arduino microcontroller were established on a standard breadboard following the manufacturer’s specifications (see Figure S4 for circuit layout and wiring details).

### 2.5. Data Acquisition and Analysis

Temperature data were acquired using an Arduino microcontroller (Arduino, Monza, Italy) programmed via the open-source Arduino Integrated Development Environment (IDE). Communication between the DS18B20 sensors and the Arduino was established through the OneWire.h [33] and DallasTemperature.h [34] libraries, which manage the 1-Wire^®^ protocol and convert raw sensor signals into calibrated temperature values (°C). Eight independent sensor instances were defined in the code, corresponding to the respective data lines. Temperature readings were retrieved at a baud rate of 9600 and printed as serial outputs, formatted with semicolon separators for structured data export. The complete Arduino code and circuit diagrams are available in previous open-source repositories [35,36,37].

For data logging and processing, a Python-based workflow (Figure S5) was implemented using the PySerial module [38]. The script continuously read serial outputs from the Arduino, appended timestamps, and stored data in Excel files with predefined column headers representing sensor positions (e.g., Living Top, Control Bottom, Incubator). To capture temperature reference values from the surrounding environment, readings from an external Temperature Phidget were acquired in parallel using the manufacturer’s algorithm [39]. The script automatically generated a new file once the current file reached its entry limit, resulting in sequential weekly datasets [37].

Subsequent data analysis was performed using a custom Python (v3.13.2) script deposited in the KIT code repository [37]. Recorded datasets were merged, time-stamped, and split by year, month, and day, as well as by quarters of the day–night illumination cycle (D1, D2, N1, N2). For each interval, mean temperature values and standard deviations were computed for all sensors. Temperature differences between corresponding sensors in living and control columns were then averaged and plotted to visualize temporal and day–night temperature variations associated with microbial thermogenesis.

### 2.6. Statistical Evaluation and Visualization

Statistical analysis was performed to assess whether temperature differences between living and control Winogradsky columns were significant. Because the datasets consisted of large, continuous time series, direct comparison of all data points resulted in artificially low *p*-values. To obtain statistically meaningful comparisons, temperature difference values were grouped into 0.01 °C intervals ranging from –1 °C to +1 °C (Figure S6). The number of occurrences within each interval was counted and normalized to the total dataset size, generating a vector of relative frequencies representing the probability distribution of temperature differences. This distribution was then subjected to non-parametric testing using the Mann–Whitney U test (alternative = “two-sided”) implemented in the scipy.stats.mannwhitneyu function [40]. The resulting *p*-values were used to evaluate whether the observed distributions of temperature differences in living versus control columns were statistically distinct. Data visualization and plotting of relative frequency distributions were performed in Python [37] using matplotlib and pandas libraries.

## 3. Results and Discussion

### 3.1. Theoretical Estimation of Microbial Heat Generation in Winogradsky Columns

To estimate the upper limit of the expected temperature rise in Winogradsky columns due to microbial metabolic activity, the temperature change rate was predicted for an idealized column colonized only by *E. coli* grown either aerobically or anaerobically. *E. coli* was selected as a well-characterized model organism with reliably documented metabolic heat yields under both conditions, allowing for a robust theoretical estimate of microbial heat generation. The purpose of this calculation was to provide an approximate upper boundary for potential temperature change within the column rather than a comprehensive comparison among different microbial species or pathways.

The temperature change rate of the sediment ($∆{\dot{T}}_{s})$ was estimated using the relation

$$\dot{Q_{b}}=m_{s}c_{s}∆{\dot{T}}_{s}$$

where $\dot{Q_{b}}$ is the heat rate produced per cell (0.8 pW for *E. coli* grown aerobically, and 0.2 pW for those grown anaerobically) [41], $m_{s}$ is the sediment mass, and $c_{s}$ is the sediment heat capacity. The cell abundance per gram of sediment was derived from literature data for environmental *E. coli* populations [42].

Detailed derivations (Supplementary Figure S7 and associated discussion) yield:

(1) $$\Delta {\dot{T}}_{sediment}=\frac{4.58}{c_{s}}{}^{\circ}C/s\;(\mathrm{aerobic})$$

(2) $$\Delta {\dot{T}}_{sediment}=\frac{1.15}{c_{s}}{}^{\circ}C/s\;(\mathrm{anaerobic})$$

The specific heat capacity of sediments typically ranges from 910 to 2400 J kg^−1^ °C^−1^, depending on composition and water content (Table S2). Based on the experimentally determined water content of 62% (Section 2.1) and literature values for dry sediment and water [43,44,45], the sediment heat capacity was estimated to range between 2880 and 4180 J kg^−1^ °C^−1^ (Figure S7, Supplementary Discussion SD2). Substituting these values into Equations (1) and (2) yields predicted heating rates of 3.9–5.6 °C h^−1^ for *E. coli* grown aerobically and 0.9–1.3 °C h^−1^ for *E. coli* grown anaerobically, corresponding to a theoretical vertical temperature difference of approximately 3–4 °C between the top and bottom of the column within one hour.

While these values lie well within the resolution of the DS18B20 sensors (±0.5 °C), they represent idealized upper limits that do not account for conductive and convective heat losses to the surrounding environment. Consequently, the experimental focus was placed on detecting consistent, smaller temperature differences between living and sterilized control columns, which would nonetheless provide direct evidence of microbial thermogenesis.

### 3.2. Short-Term Temperature Dynamics in Living and Control Columns

**Figure 3:**
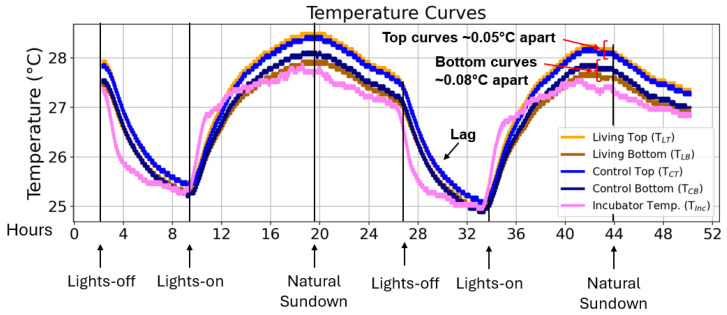
Short-term temperature profiles of living and control Winogradsky columns. Representative temperature curves recorded over a 48-h period covering two day–night illumination cycles. The plots show the temperature at the top and bottom sediment layers of the living and control columns (Abbreviations: T_LT_ = living top, T_LB_ = living bottom, T_CT_ = control top, T_CB_ = control bottom) together with the incubator temperature (T_Inc_). The *y*-axis represents absolute temperature (°C), and the *x*-axis represents time (hours). Average temperature offsets between living and control columns are indicated for the top and bottom regions, as well as the temporal lag in column temperature response following rapid incubator temperature changes.

Temperature measurements were continuously recorded over a 48-h period and visualized using the matplotlib package in Python [46]. The resulting profiles of $T_{LT}$ $T_{LB}$, $T_{CT}$, $T_{CB}$, and $T_{inc}$ revealed stable and reproducible temperature trends for both columns (Figure 3). All column temperatures followed the diurnal cycle of the incubator with a slight temporal lag during rapid temperature transitions, most notably between hours 24 and 28. This delay reflects the thermal capacitance of the sediment matrix and the plexiglass column walls, which moderate heat exchange with the environment. As verified in Supplementary Discussion SD3 in the context of Figures S8 and S9, the lag was not observed in DS18B20 sensors placed outside the columns, confirming that it was not caused by the sensor’s response time but rather by the physical properties of the sediment system.

Average temperatures in the top region of the living column were marginally higher (0.05 ± 0.05 °C) than in the control (compare $T_{LT}$ to $T_{CT}$, yellow vs. light blue curve in Figure 3). Conversely, the bottom region of the living column was slightly cooler (0.08 ± 0.08 °C) than its control counterpart (compare $T_{LB}$ to $T_{CB}$, brown vs. dark blue curve in Figure 3). These differences lie within the accuracy range of the DS18B20 sensors (±0.5 °C) [47] and are therefore not statistically significant. Variations below this threshold likely reflect sensor-specific offsets rather than biological effects.

No distinct temperature pattern differentiated the living from the control column within this initial 48-h period, indicating that short-term thermal fluctuations are primarily governed by environmental rather than microbial factors. To explore whether cumulative or periodic temperature differences might emerge over longer timescales, temperature data were subsequently averaged and analyzed over six months.

### 3.3. Vertical Temperature Gradients Within and Around the Columns

Throughout the measurement period, the upper sediment layers of both columns (T_LT_ and T_CT_; yellow and light blue curves, respectively) consistently exhibited higher temperatures than the corresponding bottom layers (T_LB_ and T_CB_; brown and dark blue curves) (Figure 3). This persistent top–bottom temperature difference suggested the possibility of localized heat generation by aerobic microorganisms in the upper region of the living column, where oxygen is more abundant. On average, the internal temperature gap in the living column (0.40 ± 0.14 °C) was slightly larger than that in the control (0.27 ± 0.05 °C). However, such differences could also arise from minor sensor offsets or from vertical temperature gradients within the incubator when the illumination was active.

**Figure 4:**
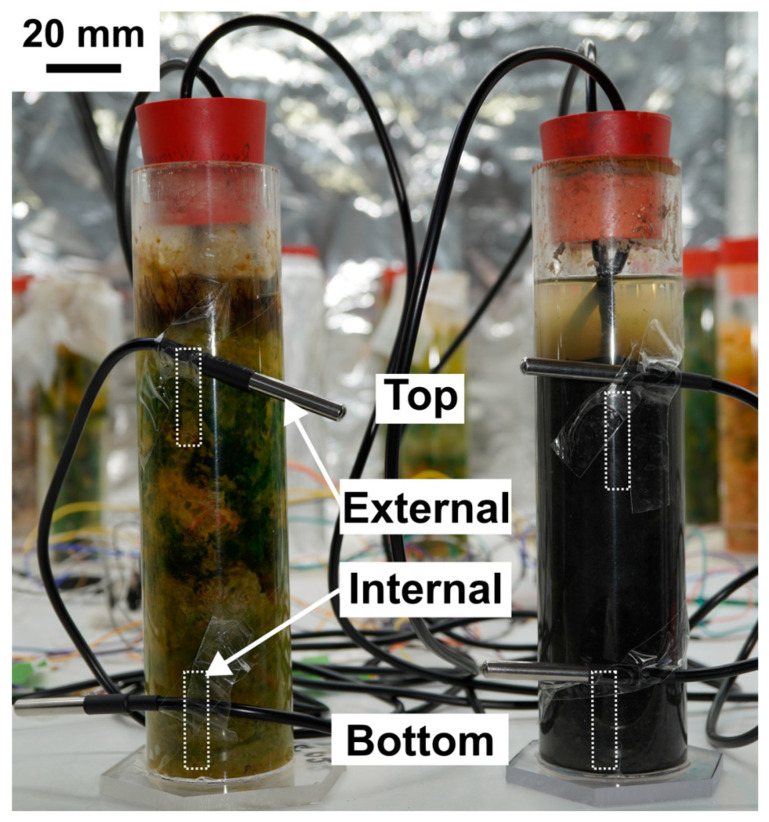
Experimental setup for measuring internal and external temperature gradients. Schematic representation of the Winogradsky column setup equipped with four additional DS18B20 sensors positioned externally to record incubator temperatures adjacent to the internal probes. The estimated locations of the internal sensors within the sediment are indicated by small dashed rectangles. External sensors were attached to the column walls at equivalent heights (~8 cm and ~18 cm below the neck) to enable direct comparison of internal and external temperature gradients. Scale bar is 20 mm.

To distinguish between biologically driven and environmental effects, additional DS18B20 sensors were positioned externally along the columns to record the incubator temperature at heights corresponding to the internal sensors (Figure 4, small dotted rectangles). The external sensors were taped to the column walls at approximately 8 cm (top) and 18 cm (bottom) below the column neck, mirroring the placement of the internal probes. These measurements enabled parallel assessment of the internal and external vertical temperature gradients, allowing evaluation of whether the observed top–bottom differences in the columns reflected intrinsic microbial heat generation or external illumination effects.

Top-to-bottom temperature gradients (ΔT_TB_) were calculated as the difference between the top and bottom sensor readings within each column, i.e., ΔT_TB–Int_ = T_LT_ − T_LB_ for the living column and ΔT_TB–Int_ = T_CT_ − T_CB_ for the control (Figure S8, see parameters ΔT_TB–Int_). External reference measurements at corresponding heights (T_LT–Ext_, T_LB–Ext_ for the living column; T_CT–Ext_, T_CB–Ext_ for the control) were used to determine the incubator’s vertical temperature gradient (ΔT_TB–Ext_). As shown in Figure S9A, all external temperature profiles overlapped closely with each other and with the incubator reference, confirming that any vertical temperature differences within the columns arose independently of ambient gradients. Minor fluctuations were observed in the external temperature profiles (Figure S9A, “Fluctuations”) and are likely attributable to ambient disturbances, such as the periodic switching of laboratory lights. These variations were not reflected in the internal temperature readings (Figure S9B), indicating that external fluctuations had no measurable influence on the thermal behavior of the columns.

To capture dynamic responses over the 24-h illumination cycle, the temperature data were divided into defined day and night phases based on the incubator’s programmed lighting schedule (see Figure S9B and Supplementary Discussion SD3). Transitional periods corresponding to sundown (T1, 00:00–01:00) and sunrise (T2, 07:00–08:00) were excluded to avoid confounding effects during light intensity changes. The remaining intervals comprised two night-phases (N1, 01:00–04:00; N2, 04:00–07:00) and two day-phases (D1, 08:00–15:30; D2, 15:30–00:00).

**Figure 5:**
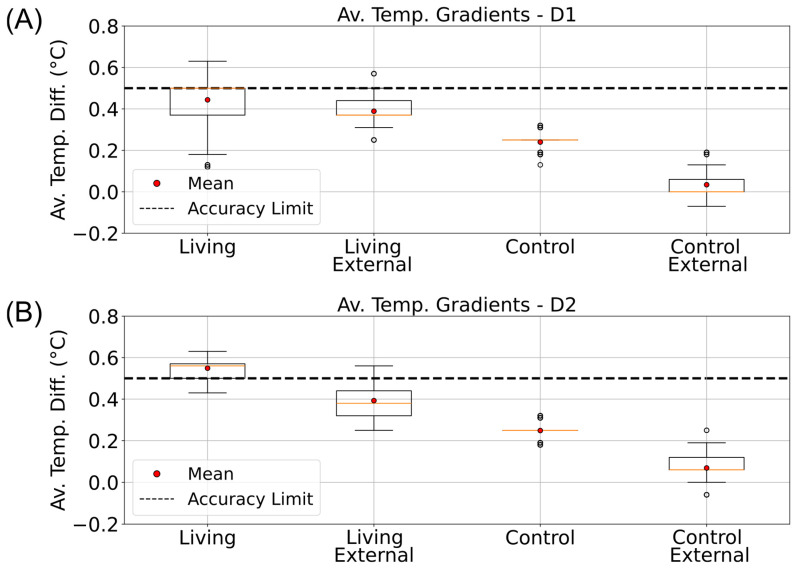
Daytime vertical temperature gradients in living and control Winogradsky columns. Boxplots showing the average top-to-bottom temperature differences (ΔT_TB_) measured inside (Internal) and outside (External) the living and control columns during the illuminated phases of the incubator’s day–night cycle. Data are arranged from left to right as follows: internal living column, external living, internal control, and external control. (**A**) Gradients calculated for the D1 phase (08:00–15:30). (**B**) Gradients calculated for the D2 phase (15:30–00:00). The dashed line indicates the ±0.5 °C accuracy range of the DS18B20 temperature sensors.

**Figure 6:**
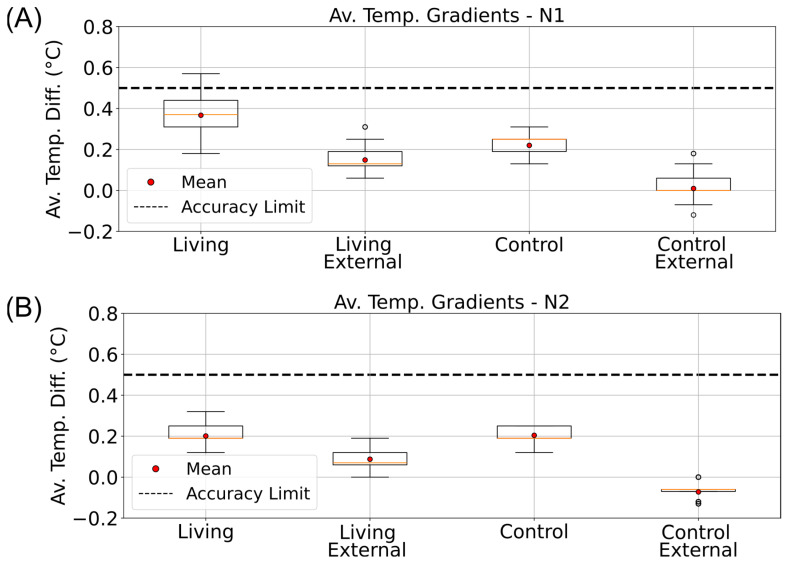
Nighttime vertical temperature gradients in living and control Winogradsky columns. Boxplots showing the average top-to-bottom temperature differences (ΔT_TB_) measured inside (Internal) and outside (External) the living and control columns during the dark phases of the incubator’s day–night cycle. Data are arranged from left to right as follows: internal living column, external living, internal control, and external control. (**A**) Gradients calculated for the N1 phase (01:00–04:00). (**B**) Gradients calculated for the N2 phase (04:00–07:00). The dashed line indicates the ±0.5 °C accuracy range of the DS18B20 temperature sensors.

Temperature gradients were computed and organized using the pandas data analysis library in Python [48]. Average ΔT_TB_ values were then calculated for each phase and visualized as boxplots (matplotlib) to compare internal and external gradients between the living and control columns (Figure 5 and Figure 6). During the illuminated phases, particularly in D2, the living column exhibited a consistently higher internal gradient (0.55 ± 0.04 °C) than both the control and its corresponding external gradient (Figure 5B). This difference exceeded the sensor’s accuracy threshold (±0.5 °C) and was not observed under dark conditions, suggesting that additional heat was produced in the oxygen-rich upper sediment layer. These findings point toward a localized temperature increase in the aerobic zone of the living column, consistent with microbial metabolic heat generation rather than passive warming from the incubator light source.

Microcalorimetric studies have demonstrated that *E. coli* releases more heat during aerobic respiration than under anaerobic fermentation, consistent with the higher per-cell heat rates used in our theoretical model (0.8 pW vs. 0.2 pW) [41,49]. Similarly, flow-calorimetric analyses of soil samples have shown that overall heat production is greater under aerobic than anaerobic conditions [50]. These findings align with our observation that the living column displayed a larger average top-to-bottom temperature gradient than the control, suggesting enhanced heat generation in the oxygen-rich upper region. Such a pattern is compatible with microbial thermogenesis associated with aerobic metabolism.

Nevertheless, this interpretation must be approached with caution. Several experimental factors could contribute to apparent temperature differences. The upper external sensor near the living column was positioned at a slightly different angle than the others (Figure 4), which may have introduced a minor bias. This effect is visible in the external temperature profiles (Figure S9A), where the living bottom trace (dark green) deviates slightly from the remaining external curves. Furthermore, thermal inertia within the sediment and the plexiglass walls could amplify transient temperature offsets. When the incubator temperature drops sharply, the column temperature decays more slowly, potentially leading to elevated internal temperature averages during these intervals.

Taken together, the data suggest that the increased temperature gradient in the living column may reflect genuine microbial metabolic activity in the upper aerobic zone, but this cannot be unambiguously distinguished from subtle experimental or thermal effects. Future refinements such as improved sensor alignment, differential calibration, and replication across multiple columns will be necessary to confirm whether the observed gradients represent a reproducible thermal signature of microbial life.

### 3.4. Long-Term Temperature Differences Between Living and Control Columns

A persistent temperature deviation between the living and control columns, even if small, could indicate continuous microbial metabolic activity. Therefore, subsequent analyses focused exclusively on temperature differences between both systems. The term temperature difference (ΔT) here refers to the difference between corresponding regions of the living and control columns: the top temperature difference (ΔT_Top_ = T_LT_ − T_CT_; see Figure S10, ΔT_Top_) and the bottom temperature difference (ΔT_Bott_ = T_LB_ − T_CB_; see Figure S10, ΔT_Bott_).

**Figure 7:**
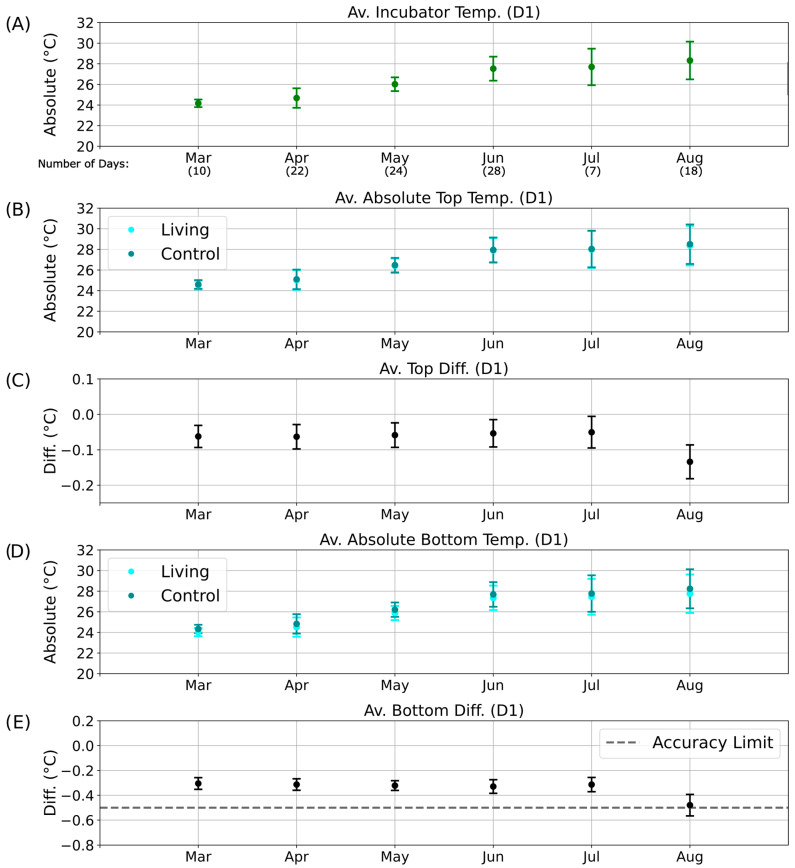
Long-term temperature analysis for the D1 phase of the day–night cycle. Average temperatures and temperature differences recorded over six months during the D1 illumination phase. The *x*-axis indicates the corresponding months of data collection, including the number of valid measurement days without technical interruptions. (**A**) Mean incubator temperature. (**B**) Average absolute temperature of the top regions of the living (cyan) and control (dark cyan) columns. (**C**) Mean top temperature difference (ΔT_Top_) between living and control columns. (**D**) Average absolute temperature of the bottom regions of the living (cyan) and control (dark cyan) columns. (**E**) Mean bottom temperature difference (ΔT_Bott_) between living and control columns.

To obtain an overview of these long-term patterns and their potential relationship with environmental temperature, monthly averages of incubator temperature during the D1 phase were computed using Python and plotted over a six-month period (Figure 7A). Corresponding average absolute temperatures for the top regions of the living and control columns are shown in Figure 7B (cyan and dark cyan, respectively), followed by their mean top temperature differences (Figure 7C). Equivalent analyses were conducted for the bottom regions (Figure 7D,E).

**Figure 8:**
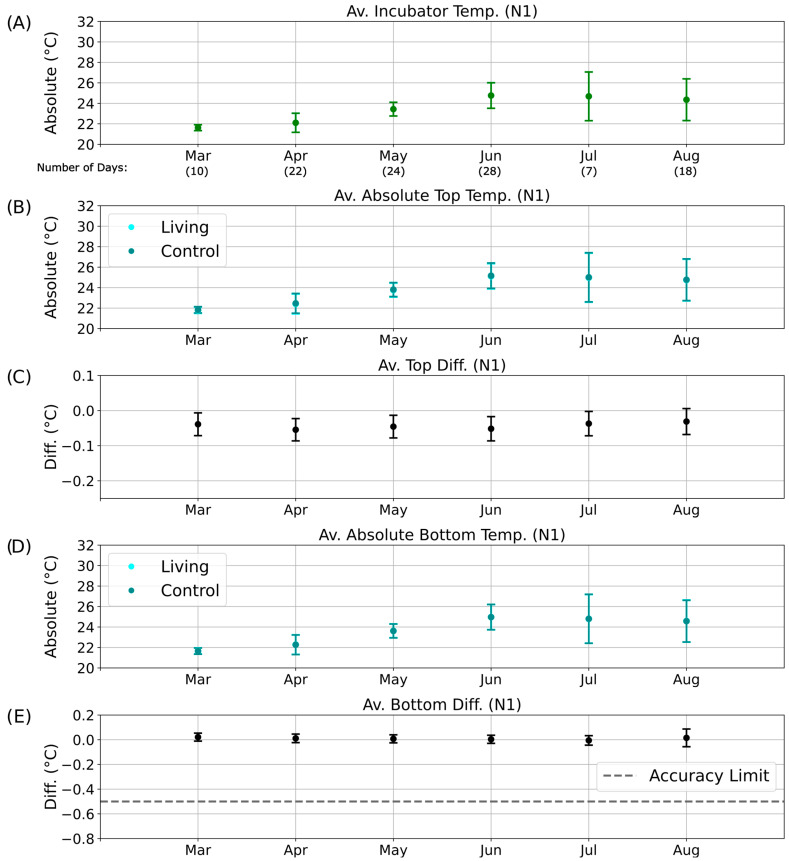
Long-term temperature analysis for the N1 phase of the day–night cycle. Average temperatures and temperature differences recorded over six months during the N1 nighttime phase. The *x*-axis indicates the corresponding months of data collection, including the number of valid measurement days without technical interruptions. (**A**) Mean incubator temperature. (**B**) Average absolute temperature of the top regions of the living (cyan) and control (dark cyan) columns. Data points for the living column overlap with those of the control column and are therefore not visible. (**C**) Mean top temperature difference (ΔT_Top_) between living and control columns. (**D**) Average absolute temperature of the bottom regions of the living (cyan) and control (dark cyan) columns. Data points for the living column overlap with those of the control column and are therefore not visible. (**E**) Mean bottom temperature difference (ΔT_Bott_) between living and control columns.

Parallel evaluations were performed for the other illumination phases: D2 (Figure S11), N1 (Figure 8), and N2 (Figure S12). These datasets collectively allowed comparison of temperature differences across diurnal and seasonal scales, providing insight into whether consistent deviations could be linked to biological heat production rather than external environmental variation.

Because of occasional technical interruptions, continuous temperature data were not available for all days of each month. For months with sufficient data coverage (April to June; Figure 7 and Figure 8), the temperature differences between the living and control columns remained stable despite fluctuations in incubator temperature, and no patterns indicative of metabolic activity were detected. By August, however, the bottom temperature difference had approached the sensor accuracy limit (Figure 7E), and the associated error bars exceeded this threshold. This anomaly may indicate the onset of biological activity in the control column, potentially resulting from gradual air leakage through the rubber stopper. Although designed to be airtight, the stoppers may have permitted the slow ingress of oxygen, facilitating colonization by aerobic or sulfate-reducing microorganisms.

During the D2 illumination phase (Figure S11E), the same deviation persisted but remained within the overall accuracy range. In contrast, during the nighttime phases (N1 and N2; Figure 8 and Figure S12), the absolute temperatures of the living and control columns were nearly identical, with overlapping data points (dark cyan obscuring cyan in Figure 8B,D and Figure S12B,D). Correspondingly, the bottom temperature difference remained close to 0 °C (Figure 8E and Figure S11E), indicating the absence of detectable metabolic heating under dark, anaerobic conditions.

Over time, visible changes were observed in both systems (Figure S13). The control column developed a darker coloration (Figure S13A), consistent with the possible growth of sulfate-reducing bacteria following minor oxygen ingress. Meanwhile, the living column exhibited partial drying and shrinkage at the top (Figure S13B), which may have disturbed its microbial community structure and reduced overall metabolic activity. As discussed in Supplementary Discussion SD4, these factors likely contributed to unexpected biological activity in the control column and a decline in activity within the living column. Overall, no reproducible long-term temperature patterns consistent with sustained microbial thermogenesis could be confirmed when comparing the living and control columns.

### 3.5. Effect of Light Shielding on Temperature Dynamics

To minimize radiative and convective heat exchange between the columns and their surroundings, both the living and control columns were temporarily wrapped in aluminum foil while temperature measurements continued. The insulation was applied during the early part of the D1 phase (see Figure S9B comparing external and internal temperature profiles and defining illumination phases for quarter intervals) and removed after 24 h.

Insulating the columns resulted in an overall increase in the temperature difference between the living and control systems. This effect, referred to as temperature retention, was quantified as ΔΔT_Top_ and ΔΔT_Bott_, defined as the difference between the highest average ΔT during insulation and the average ΔT over the preceding three days. In cases where temperature differences remained elevated after foil removal, a steady-state temperature retention (ΔΔT_Top-Steady_ and ΔΔT_Bott-Steady)_ was calculated as the difference between the post- and pre-insulation three-day averages. These parameters were used to assess whether insulation produced lasting thermal effects potentially associated with changes in microbial community activity.

**Figure 9:**
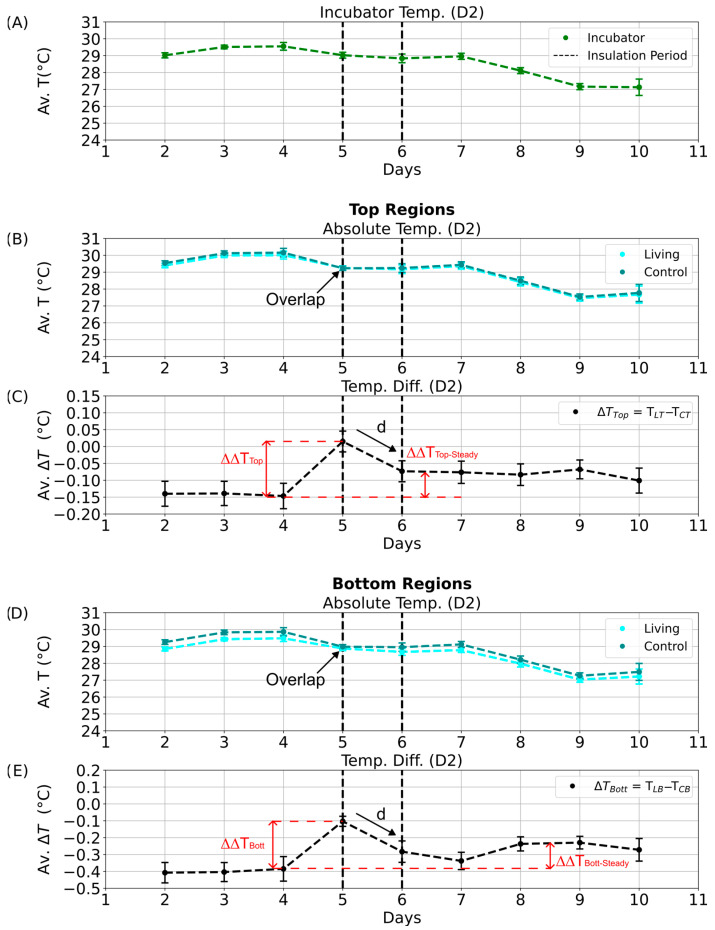
Daytime temperature dynamics during the aluminum foil insulation experiment (D2 phase). Average temperatures and temperature differences between the living and control Winogradsky columns during the D2 quarter of the day–night cycle. Error bars represent standard deviations, and black dashed lines mark the start and end of the insulation period. (**A**) Mean incubator temperature. (**B**) Average absolute temperature of the top regions of the living (cyan) and control (dark cyan) columns. (**C**) Average temperature difference between the top regions of the living and control columns. Red dashed lines denote the calculated temperature retention (ΔΔT_Top_ and ΔΔT_Bott_) and steady-state temperature difference (ΔΔT_Top-Steady_ and ΔΔT_Bott-Steady_), with arrows indicating the decline from maximum values. (**D**) Average absolute temperature of the bottom regions of the living (cyan) and control (dark cyan) columns. (**E**) Average temperature difference between the bottom regions of the living and control columns.

Temperature and retention parameters were analyzed for all illumination phases (D1, D2, N1, N2; see Figure S9B comparing internal and external temperature profiles). During D2, the most pronounced response was observed: insulation caused parallel temperature decreases in both columns, but the living column consistently retained more heat (Figure 9B,D; compare overlaps in Figure 9C,E). The corresponding increases in ΔΔT_Top_ and ΔΔT_Bott_ indicated enhanced heat retention in the living column. Although the theoretically predicted heating rate for *E. coli* grown anaerobically (0.9–1.3 °C h^−1^, see Section 3.1) was not observed under these experimental conditions, this discrepancy likely reflects the dynamic nature of microbial metabolism. In contrast to the model assumption of constant per-cell heat generation, actual heat production fluctuates with growth stage and metabolic activity, leading to lower apparent heating rates in complex sediment systems.

**Figure 10:**
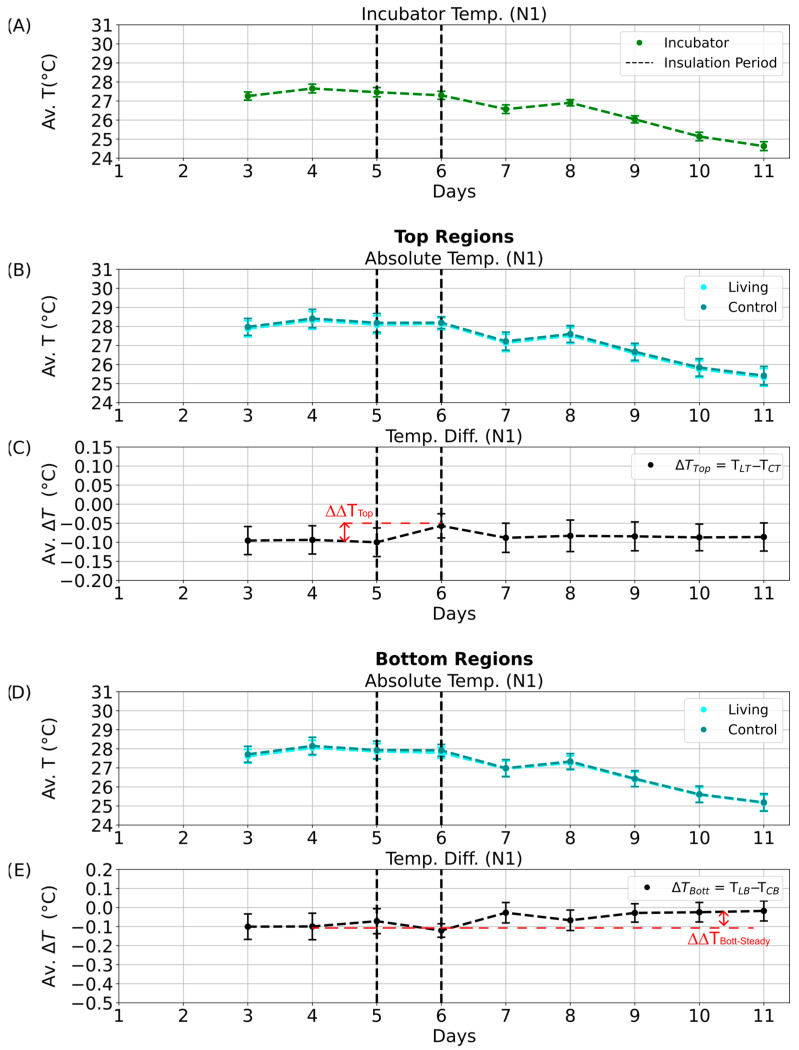
Nighttime temperature dynamics during the aluminum foil insulation experiment (N1 phase). Average temperatures and temperature differences between the living and control Winogradsky columns during the N1 quarter of the day–night cycle. Error bars represent standard deviations, and black dashed lines mark the start and end of the insulation period. (**A**) Mean incubator temperature. (**B**) Average absolute temperature of the top regions of the living (cyan) and control (dark cyan) columns. (**C**) Average temperature difference between the top regions of the living and control columns. Red dashed lines indicate the calculated temperature retention (∆∆T_Top_ and ∆∆T_Bott_) and steady-state temperature difference (∆∆T_Top-Steady_ and ∆∆T_Bott-Steady_), with arrows marking declines from the maximum values. (**D**) Average absolute temperature of the bottom regions of the living (cyan) and control (dark cyan) columns. (**E**) Average temperature difference between the bottom regions of the living and control columns.

Even after insulation was removed, the living column maintained slightly elevated temperature differences (Figure 9C,E; ΔΔT_Top-Steady_ and ΔΔT_Bott-Steady_), suggesting a transient thermal stabilization or potential microbial response. Similar patterns were detected during the D1 phase (Figure S14C,E). In contrast, no significant effects were observed during the night phases (N1, N2; Figure 10C,E and Figure S15C,E), likely due to dominant heat loss through the uninsulated column base and reduced microbial metabolic activity in the absence of light.

**Figure 11:**
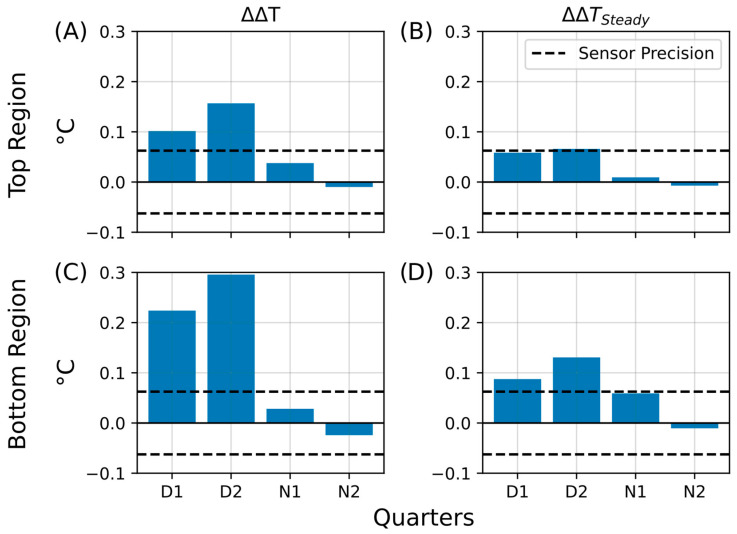
Summary of temperature retention effects during aluminum foil insulation. Bar plots summarizing key parameters that describe heat retention in the living Winogradsky column relative to the control during the first insulation experiment. The *y*-axis represents the change in temperature difference (ΔT, °C), and the *x*-axis indicates the day–night quarters (D1, D2, N1, N2) during which the changes were observed. The dashed line denotes the sensor precision (0.0625 °C). (**A**) Temperature retention (ΔΔT) in the top region. (**B**) Steady-state temperature difference after insulation (ΔΔT_Steady_) in the top region. (**C**) Temperature retention (ΔΔT) in the bottom region. (**D**) Steady-state temperature difference after insulation (ΔΔT_Steady_) in the bottom region.

In the aluminum foil shielding experiment, both the living and control Winogradsky columns were wrapped in aluminum foil to reduce heat exchange with the surrounding environment. During the insulation period, the living column retained heat more effectively than the control, as indicated by the increased temperature difference between their top regions (ΔΔT; Figure 11A). This effect was most pronounced during the illuminated D2 phase, where the ΔΔT bar exceeded those of all other phases, but it diminished substantially during the night (N1 and N2), falling below the sensor’s precision threshold. These results suggest that, under insulation, the living column exhibited enhanced heat retention, possibly associated with microbial metabolic activity in the aerobic upper layers.

Following the insulation period, the average temperature difference remained slightly elevated (ΔΔT_Steady_; Figure 11B), particularly during the daytime phases D1 and D2, where values approached the sensor’s precision limit (±0.0625 °C). At night, this effect was markedly reduced, consistent with greater heat loss under dark, cooler conditions. The bottom regions exhibited an even stronger response: the temperature retention (ΔΔT; Figure 11C) and steady-state difference (ΔΔT_Steady_; Figure 11D) both exceeded the sensor precision during D1 and D2, while being less pronounced during N1 and N2. These results imply that insulation not only limited external heat loss but may also have triggered shifts in microbial community activity, particularly in the lower, less oxygenated zones of the living column.

To verify the results of the initial insulation experiment, a second aluminum foil insulation test was performed with an extended duration. Both columns were wrapped during the D2 phase and continuously monitored for four days. Temperature data were evaluated for all four quarters of the day–night cycle (D1, D2, N1, and N2; see Figure S9B for the definition of illumination phases and comparison of internal and external temperature profiles). Absolute temperature values and corresponding differences between the living and control columns were plotted for each phase (Figures S16–S19 for D1, D2, N1, and N2, respectively).

During the D1 phase, the living column retained heat more efficiently than the control, particularly in the bottom region (Figure S16E, ΔΔT_Bottom_). This effect likely reflected sustained microbial activity from communities adapted to low-light or anaerobic conditions. After insulation was removed, the temperature profiles returned to their pre-insulation state (Figure S16C–E, “return”), indicating that the heat retention effect was transient. The extended insulation period appeared to disturb the thermal and microbial equilibrium of the system and thus prevented the living column from maintaining elevated temperatures once the foil was removed, in contrast to the more persistent effects observed in the first experiment. Similar transient effects were recorded in the D2 phase (Figure S17E).

During the night phases (N1 and N2), heat retention was limited mainly to the upper regions (Figure S18C), whereas the lower regions lost heat more rapidly (Figure S18E), likely due to incomplete insulation of the column base. By the N2 phase, the retention effect had disappeared entirely (Figure S19C–E). When comparing D1 and D2 (Figures S16C and S17C), the rise in temperature difference in the living column appeared delayed in D1 (occurring after the dashed line) but immediate in D2 (before the dashed line), suggesting that the retention response depended on the timing of insulation relative to the light cycle. A detailed analysis of these dynamics is provided in Supplementary Discussion SD5.

**Figure 12:**
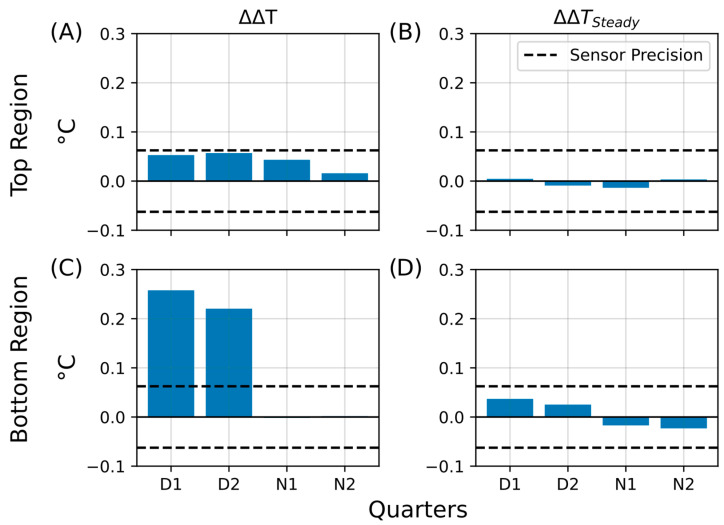
Summary of temperature retention effects during the extended aluminum foil insulation experiment. Bar plots summarizing key parameters from the second insulation experiment, in which the Winogradsky columns were covered with aluminum foil for four days. The plots show temperature retention in the living column relative to the control (ΔΔT) and persistent changes after insulation (ΔΔT_Steady_). The *y*-axis represents the change in temperature difference (ΔT, °C), and the *x*-axis indicates the quarters of the day–night cycle (D1, D2, N1, N2) during which the changes were observed. The dashed line marks the sensor precision (±0.0625 °C). (**A**) Temperature retention (ΔΔT) in the top region. (**B**) Steady-state temperature difference (ΔΔT_Steady_) in the top region. (**C**) Temperature retention (ΔΔT) in the bottom region. (**D**) Steady-state temperature difference (ΔΔT_Steady_) in the bottom region.

Bar plots summarizing the calculated temperature retention (ΔΔT) and steady-state differences (ΔΔT_Steady_) for all four phases (D1, D2, N1, N2) are presented in Figure 12. The temperature retention effect was weak in the upper regions (Figure 12A, ΔΔT_Top_), with most bars approaching the sensor precision limit. No significant steady-state increase was detected (Figure 12B, ΔΔT_Top-Steady_), as all values remained below 0.01 °C. In contrast, the bottom regions showed more pronounced retention during D1 and D2 (Figure 12C, ΔΔT_Bott_), whereas heat dissipation dominated during the night (N1 and N2), with no values exceeding –0.01 °C. Similarly, steady-state differences in the bottom region remained minimal (Figure 12D, ΔΔT_Bott-Steady_).

These results indicate that microbial metabolism in the living column partially compensated for the loss of external heat during insulation, particularly in the lower, oxygen-poor layers. The extended shielding likely suppressed phototrophic activity in the upper regions, where cyanobacteria and microscopic eukaryotic algae typically dominate, while dark-adapted microbial populations in the sediment base maintained limited thermal generation. However, the longer insulation period may also have disrupted the natural balance of the microbial communities, preventing the community shifts observed during the initial short-term insulation experiment.

**Figure 13:**
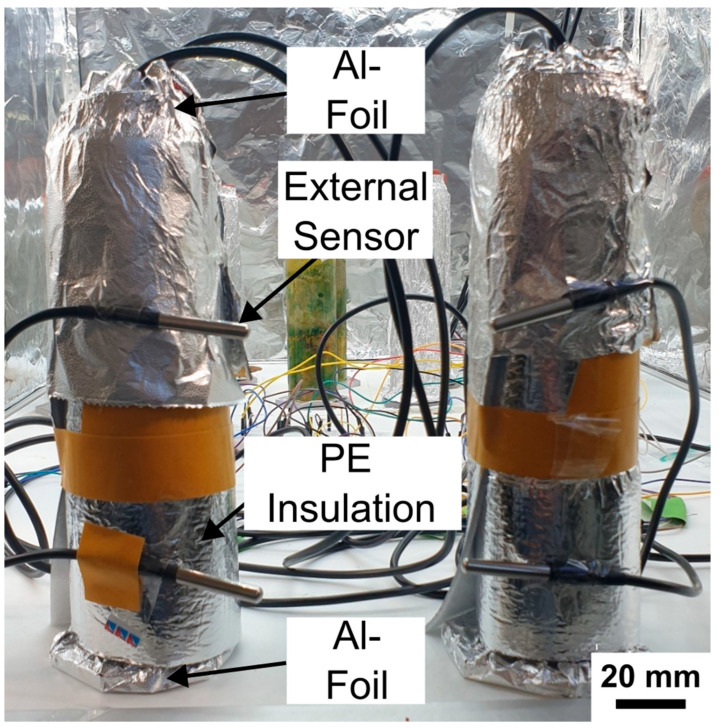
Experimental setup for the third insulation experiment using polyethylene and aluminum foil. Both Winogradsky columns were tightly wrapped with insulation sheets composed of aluminum foil lined with polyethylene foam. Additional aluminum foil layers were applied to the top and bottom ends to ensure complete thermal coverage. External DS18B20 sensors were attached to the surface of the wrapped columns to monitor the incubator temperature adjacent to each column and to assess potential external influences on internal temperature dynamics. Scale bar is 20 mm.

As described previously, both columns had experienced water loss due to evaporation. To restore comparability between the systems, deionized water was added to each column to equalize the height of the supernatant. A third insulation experiment was then conducted using a double-layer setup consisting of polyethylene (PE) insulation wrapped in aluminum foil (doitBau GmbH & Co. KG, Wuppertal, North Rhein-Westphalia, Germany; see Figure 13 for setup). Because of the thickness and rigidity of the insulation material, complete coverage of the column base and rubber stopper was not feasible. To minimize remaining thermal exchange, the upper and lower ends of both columns were therefore additionally wrapped with an extra layer of aluminum foil (Figure 13, “Al-Foil”).

**Figure 14:**
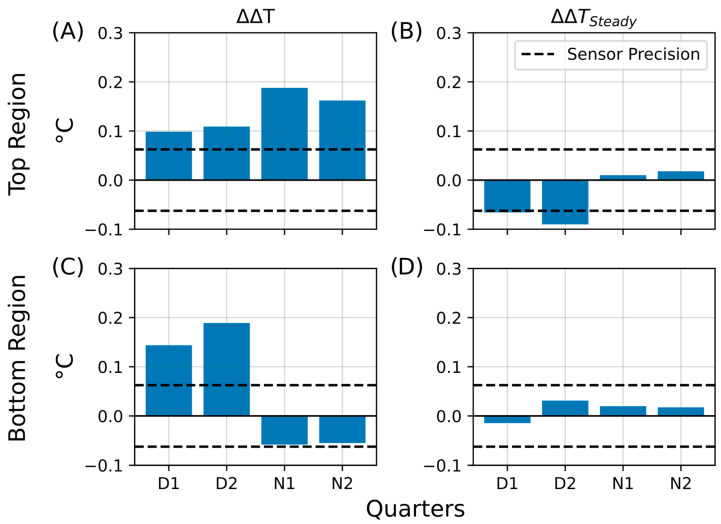
Summary of temperature retention effects during the third insulation experiment (PE and aluminum foil). Bar plots summarizing key parameters from the third insulation experiment, in which the Winogradsky columns were wrapped with polyethylene (PE) insulation and aluminum foil. The plots show temperature retention in the living column relative to the control (ΔΔT) and persistent changes after insulation (ΔΔT_Steady_). The *y*-axis represents the change in temperature difference (ΔT, °C), and the *x*-axis indicates the quarters of the day–night cycle (D1, D2, N1, N2) during which the changes were observed. The dashed line marks the sensor precision (±0.0625 °C). (**A**) Temperature retention (ΔΔT) in the top region. (**B**) Steady-state temperature difference (ΔΔT_Steady_) in the top region. (**C**) Temperature retention (ΔΔT) in the bottom region. (**D**) Steady-state temperature difference (ΔΔT_Steady_) in the bottom region.

The third insulation experiment, using combined aluminum–polyethylene (Al–PE) wrapping, further confirmed the transient nature of the heat retention effects. Initially, the living column retained slightly more heat than the control, particularly in the bottom region, but this difference diminished over time and reversed after prolonged dark conditions. During the nighttime phases, the living column consistently exhibited slightly higher top-region temperatures than the control, suggesting residual microbial activity adapted to dark cycles. Figure 14 summarizes the main results of this experiment, showing temperature retention (ΔΔT) and steady-state differences (ΔΔT_Steady_) for all four illumination phases (D1, D2, N1, and N2). The data reveal that transient heat retention occurred primarily during the illuminated phases, while extended dark conditions led to a return to baseline temperature differences. A detailed phase-resolved analysis, including complete temperature profiles and ΔΔT calculations, is provided in the Supplementary Information (Figures S20–S23 and Supplementary Discussion SD6).

## 4. Conclusions

Temperature is a fundamental determinant of microbial activity, shaping both metabolic rates and community organization. In this study, we developed and applied a low-cost, continuous temperature sensing system directly integrated into living Winogradsky columns to assess temperature as a non-invasive proxy for microbial metabolism. By estimating the sediment heat capacity, we derived theoretical upper limits for microbial heat generation and experimentally demonstrated localized thermal gradients consistent with aerobic metabolic activity.

The results establish temperature sensing as a viable, real-time indicator of microbial processes in complex sediment ecosystems. Future improvements may include the use of smaller sensors for radial or depth-resolved measurements, integration of higher-precision sensors via printed circuit boards (PCBs) with waterproof encapsulation, and coupling of the temperature data with complementary electrochemical or optical sensors. Such developments could enhance spatial resolution, sensitivity, and long-term stability of the system.

Overall, the methods and analytical framework presented here provide a foundation for extending low-cost digital temperature sensing to the study of dynamic microbial ecosystems and for advancing non-invasive environmental monitoring technologies.

## Data Availability

Excel outputs containing temperature readings along with Python code used for data processing are found on the KIT repository [37]. Other data available upon reasonable request from the corresponding author.

## Supplementary Materials

The following supporting information can be downloaded at: https://www.mdpi.com/article/10.3390/s25237146/s1, Table S1: Resulting masses of sediment samples pre- and post- drying, and the calculated ratio dry-to-fresh sediment ratio in %; Table S2: Specific heat capacities of different types of sediment found in literature; Figure S1: Photographic image of an empty PMMA cylinder used to prepare Winogradsky columns; Figure S2: External view of incubator constructed to position the columns under a 24-hour day-night cycle. B) Inside of incubator showing two Winogradsky columns with integrated temperature sensors, as well as LED programmable illumination; Figure S3: Schematic and configuration of the DS18B20 temperature sensor; Figure S4: Circuit diagram and breadboard implementation for DS18B20 sensor integration; Figure S5: Flowchart of data acquisition and analysis workflow; Figure S6: Statistical analysis of temperature difference distributions; Figure S7: Conceptual model of heat generation by *E. coli* in hypothetical Winogradsky columns; Figure S8: Measurement parameters used for vertical temperature comparison; Figure S9: Comparison of external and internal temperature profiles and definition of illumination phases; Figure S10: Illustration of the parameters used to investigate temperature differences over months; Figure S11: Long-term temperature analysis for the D2 phase of the day–night cycle; Figure S12: Long-term temperature analysis for the N2 phase of the day–night cycle; Figure S13: Physical changes in the Winogradsky columns during the course of the experiment; Figure S14: Daytime temperature dynamics during the aluminum foil insulation experiment (D1 phase); Figure S15: Nighttime temperature dynamics during the aluminum foil insulation experiment (N2 phase); Figure S16: Daytime temperature dynamics during the second aluminum foil insulation experiment (D1 phase); Figure S17: Daytime temperature dynamics during the second aluminum foil insulation experiment (D2 phase); Figure S18: Nighttime temperature dynamics during the second aluminum foil insulation experiment (N1 phase); Figure S19: Nighttime temperature dynamics during the second aluminum foil insulation experiment (N2 phase); Figure S20: Daytime temperature dynamics during the third insulation experiment (D1 phase); Figure S21: Daytime temperature dynamics during the third insulation experiment (D2 phase); Figure S22: Nighttime temperature dynamics during the third insulation experiment (N1 phase); Figure S23: Nighttime temperature dynamics during the third insulation experiment (N2 phase); Discussion SD1: Derivation of Temperature Change Rates in the Hypothetical Columns; Discussion SD2: Estimating the Range of the Specific Heat Capacity; Discussion SD3: Analysis of temporal lag in column temperature profiles; Discussion SD4: Evidence of microbial growth in the control column; Discussion SD5: Analysis of temperature changes during the extended (4-day) insulation experiment; Discussion SD6: Analysis of temperature changes during the Al-PE insulation experiment. The following references were cited in the supplementary information: [41,42,43,44,45,47,51,52,53,54,55,56,57].
