# Supplementary material for: Low-Cost Temperature Sensing Reveals Thermal Signatures of Microbial Activity in Winogradsky Columns
Source: Sensors (Basel). 2025 Nov 22;25(23):7146. doi: 10.3390/s25237146 (PMC12693836; doi:10.3390/s25237146)
Supplement: Supplementary file 1 [file sensors-25-07146-s001.zip › sensors-3968418-supplementary.pdf]

Low-cost temperature sensing reveals thermal signatures of microbial activity in Winogradsky columns

Ahmad Itani,[1] Dario Mager,[2] Kersten S. Rabe,[1] Christof M. Niemeyer*[1]

[1] Karlsruhe Institute of Technology (KIT), Institute for Biological Interfaces 1 (IBG-1), Biomolecular Micro- and Nanostructures, Hermann-von-Helmholtz-Platz 1, D-76344 Eggenstein-Leopoldshafen, Germany.

[2] Karlsruhe Institute of Technology (KIT), Institute of Microstructure Technology (IMT), Hermann-von-Helmholtz-Platz 1, D-76344 Eggenstein-Leopoldshafen, Germany.

Supplementary information

Supporting Tables

Table S1. Resulting masses of sediment samples pre- and post- drying, and the calculated ratio dry-to-fresh sediment ratio in %.

Sample No.	Starting Mass (g)	After 12 hours drying	After 24 hours drying	% Dry sediment
1	30	11.2	11.2	37.3
2	30	11.1	11.1	37
3	30	10.9	10.9	36.3
4	30	11.2	11.2	37.3
5	30	11	11	36.7
6	20	7.7	7.7	38.5
7	20	8.1	8.1	40.5
8	40	16.2	16.2	40.5
9	40	15.8	15.8	39.5

Table S2. Specific heat capacities of different types of sediment found in literature. A dash (-) indicates that the information was not available in the indicated source.

Source	c_s (J/Kg°C)	Type of Sediment	Water Content (kg/kg)
Zhu, *et al.* [51]	1350	Mud	-
Goto, *et al.* [52]	2400	Mud	-
Abu-Hamdeh [43]	1200-2400	Clay Soil	0.02-0.25 (kg/kg)
	800-1600	Sandy Soil	0.02-0.25 (kg/kg)
Ghuman and Lal [53]	910	Sandy Soil	-
	1350	Clay Soil	-
Yadav and Saxena [54]	810-1610	Sandy Soil	-
	1130-1980	Clay Soil	-
Wang, *et al.* [44]	750	Dry Sediment	-
This Study	c_s	Benthic Sediment	62%

Supporting Figures

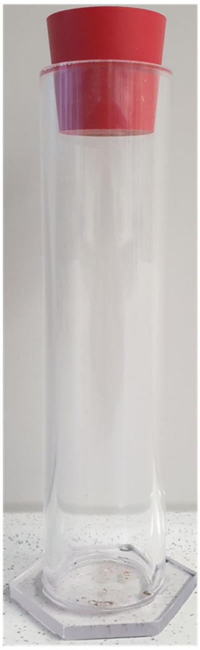

Figure S1. Photographic image of an empty PMMA cylinder used to prepare Winogradsky columns.

A)

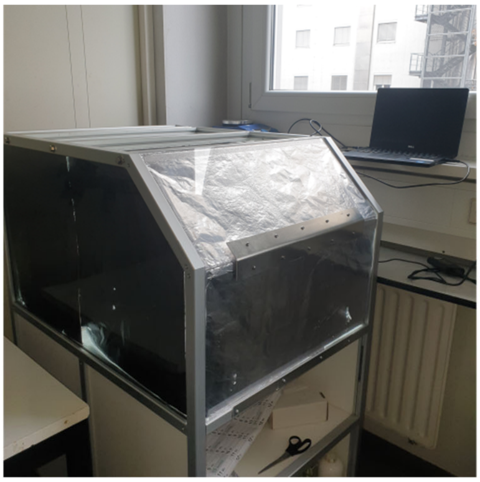

B)

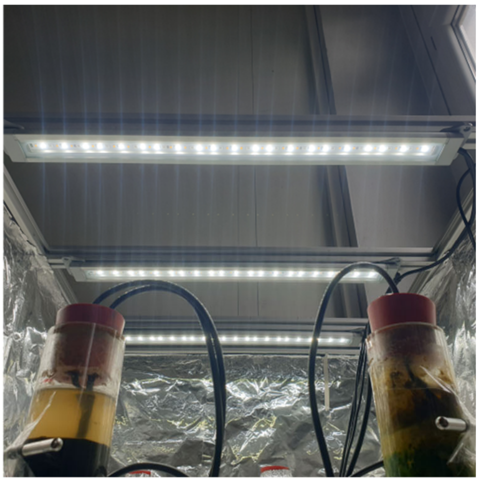

Figure S2. A) External view of incubator constructed to position the columns under a 24-hour day-night cycle. B) Inside of incubator showing two Winogradsky columns with integrated temperature sensors, as well as LED programmable illumination.

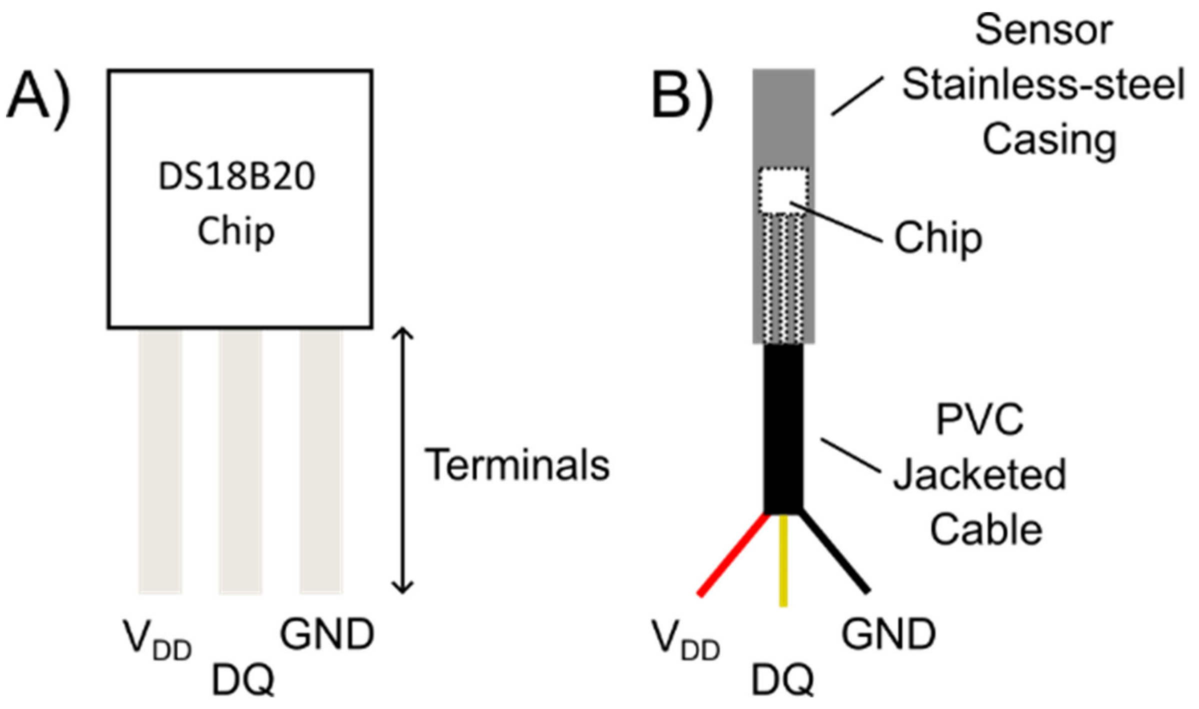

Figure S3. Schematic and configuration of the DS18B20 temperature sensor. A) Diagram of the DS18B20 sensor chip indicating the three terminals: power supply (VDD), data line (DQ), and ground (GND). B) Illustration of the waterproof DS18B20 probe encapsulated in a stainless-steel housing, with the terminals connected through a polyvinyl chloride (PVC) cable and exposed at the distal end for connection to the microcontroller.

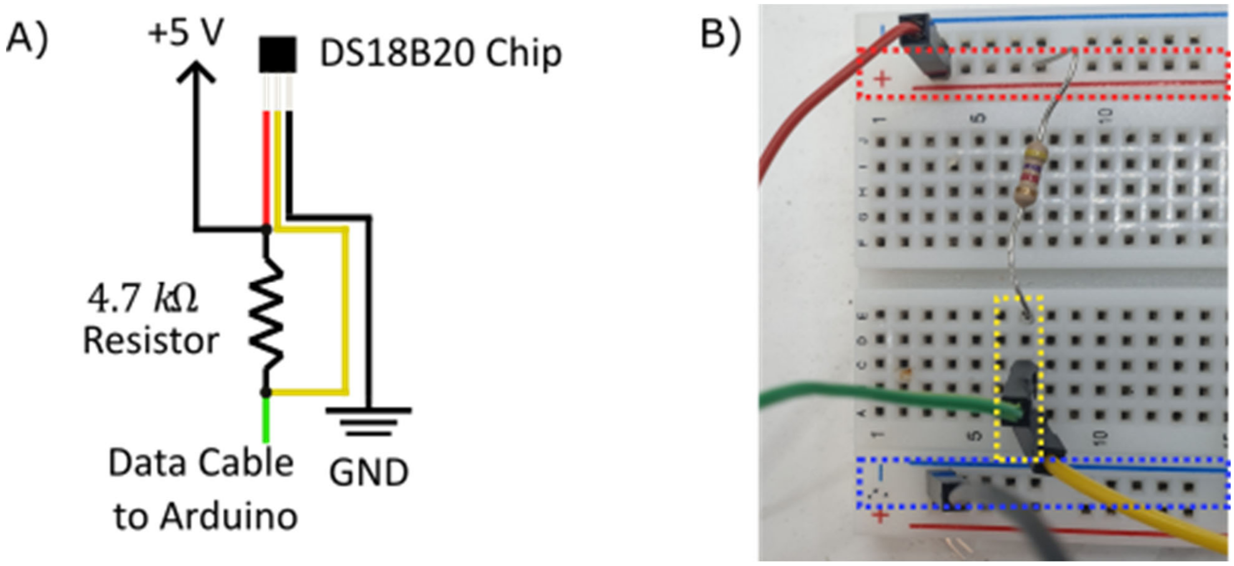

Figure S4. Circuit diagram and breadboard implementation for DS18B20 sensor integration. A) Schematic representation of the circuit showing the required connections between the Arduino microcontroller and the DS18B20 sensors. The setup includes a 4.7 kΩ pull-up resistor between the data line and the 5 V power rail to maintain a stable high state when the sensors are idle.[47] B) Photograph of the assembled circuit on a breadboard (Frei GmbH). Each row (yellow dashed box) represents a shared connection rail. The red and blue columns indicate the 5 V power and ground rails, respectively. The sensor power cable (red) and ground cable (black) are connected to these rails, while the data cable (yellow) is linked to one of the Arduino's digital input pins through the data rail. Two sensor circuits are shown as implemented in this study.

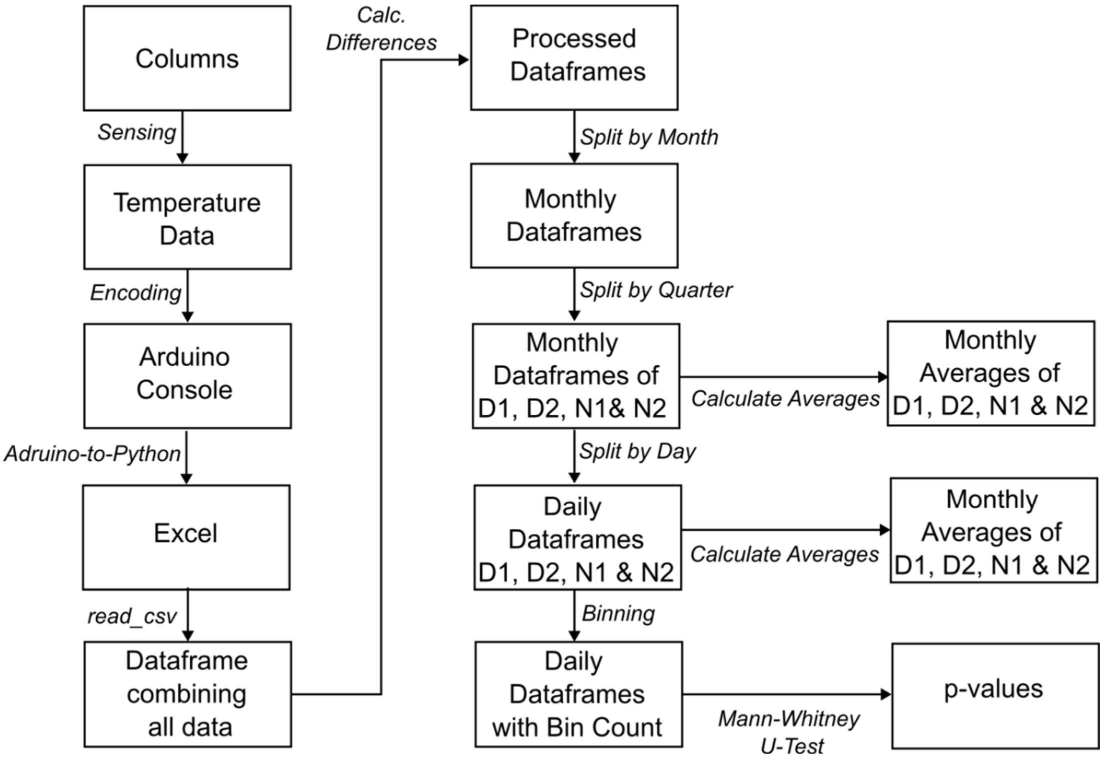

Figure S5. Flowchart of data acquisition and analysis workflow. Flowchart summarizing the algorithm used in the Python code to process temperature sensor data recorded in the Winogradsky columns. Temperature readings from DS18B20 sensors were collected by the Arduino microcontroller via the 1-Wire® protocol and transmitted to a connected computer through serial communication. A Python script using the PySerial module automatically logged, time-stamped, and organized the data into structured Excel files with predefined column headers representing sensor positions. These files were subsequently merged and analyzed with a custom script to calculate mean temperature values, standard deviations, and day–night cycle averages, enabling visualization of temperature differences between living and control columns.

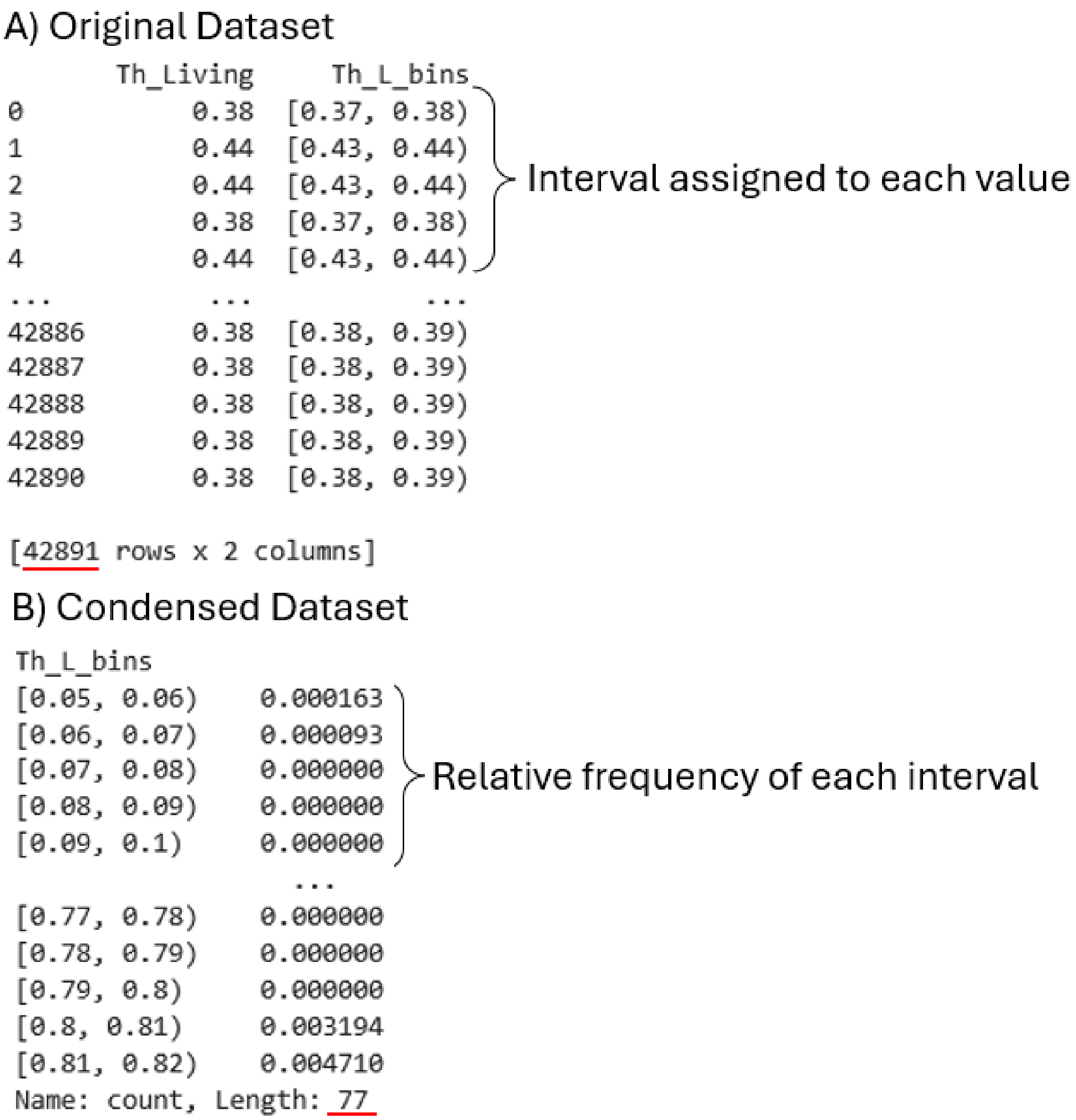

Figure S6. Statistical analysis of temperature difference distributions. A) Snapshot of the original dataset showing temperature difference values between living and control Winogradsky columns assigned to 0.01 °C bins within the range of –1 °C to +1 °C. B) Condensed dataset representing the relative frequency of values in each bin, used for subsequent statistical testing. Relative frequency distributions were compared between conditions using a two-sided Mann–Whitney U test (scipy.stats.mannwhitneyu). This analysis evaluates whether the distributions of temperature differences in living versus control columns differ significantly, indicating measurable microbial thermogenesis.

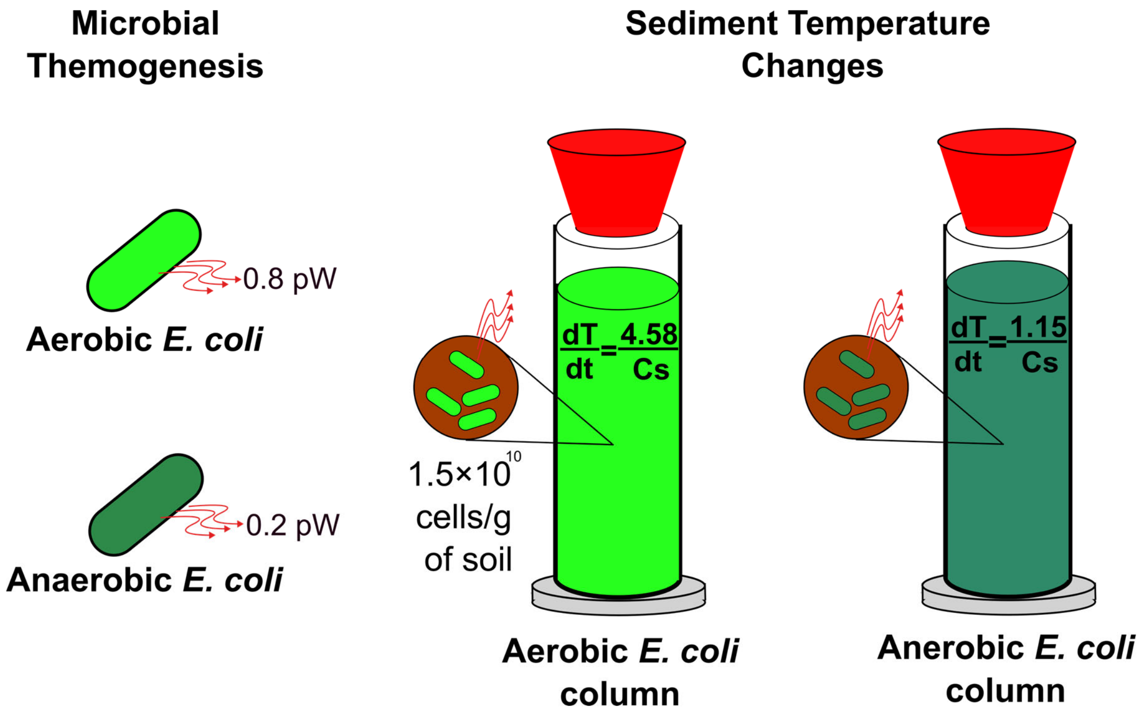

Figure S7. Conceptual model of heat generation by *E. coli* in hypothetical Winogradsky columns. Schematic illustration of two idealized Winogradsky columns modeled for theoretical estimation of microbial heat generation. Each column is assumed to be colonized by *E. coli* cells (1.5 × 10^{10} cells per gram of dry sediment).[42] The heat rate per cell varies with metabolic state: cells grown aerobically (light green) produce 0.8 pW, whereas cells grown anaerobically (dark green) produce 0.2 pW.[41] These values were used to derive the theoretical temperature change rates presented in the below discussion, taking into account the specific heat capacity (cs) of the sediment determined in this study.

Discussion SD1: Derivation of Temperature Change Rates in the Hypothetical Columns

$\Delta\dot{T}_s$ can be estimated using the formula:

$$\dot{Q}_b = m_s c_s \Delta\dot{T}_s \tag{S1}$$

$\dot{Q}_b$ is the heat rate generated in watt by all bacteria in the column, m_s is the mass of column sediment in gram, and c_s is the sediment heat capacity in J/kg°C.

$\dot{Q}_b$ can be estimated by calculating the number of bacterial cells n_b per gram of sediment, and multiplying it by the estimated heat rate generated per bacterial cell ($\dot{Q}_{cell}$):

$$\dot{Q}_b = n_b \dot{Q}_{cell} \tag{S2}$$

It is approximated that 1.50x10^{10} bacterial cells flourish per 1 g of dry soil.[42] The estimated total number of *E. coli* in the column is therefore given by:

$$n_b = 1.50 \times 10^{10} \times m_{dry} \quad \text{(S3)}$$

Where m_{dry} is the mass of dry sediment in the column in grams (g). Equation (S2) then becomes:

$$\dot{Q}_b = 1.50 \times 10^{10} \times m_{dry}\dot{Q}_{cell}$$

A single cell of *E. coli* produces heat at a rate of $\dot{Q}_{cell}$ = 0.80 pW when aerobic, and 0.20 pW when anaerobic.[41]

The total heat generated by aerobically grown *E. coli* in the column is therefore:

$$\dot{Q}_b = 1.50 \times 10^{10} \times m_{dry} \times 0.80 \quad \text{(S4)}$$

For anaerobically grown *E. Coli*, the equation becomes:

$$\dot{Q}_b = 1.50 \times 10^{10} \times m_{dry} \times 0.20 \quad \text{(S5)}$$

Rearranging Equation (S1):

$$\Delta\dot{T}_s = \frac{\dot{Q}_b}{m_s c_s} \quad \text{(S6)}$$

Plugging the variables from (S4) or (S5) in (S6), the equation becomes:

$$\Delta\dot{T}_s\ [°C/s] = \frac{1.50 \times 10^{10}[\text{cells/g}] \times m_{dry}[g]\dot{Q}_{cell}[W]}{m_s[\text{Kg}]\ \ c_s[\frac{J}{kg°C}]} \quad \text{(S7)}$$

Units are shown in brackets to track unit conversions. Changing all mass units to kg, the cell density becomes 1.50 x 10^{13} cells/kg , yielding:

$$\Delta\dot{T}_{sediment}\ [°C/s] = \frac{1.50 \times 10^{13}[\text{cells/kg}] \times m_{dry}[kg]\dot{Q}_{cell}[W]}{m_s[\text{kg}]\ \ c_s[\frac{J}{kg°C}]} \quad \text{(S8)}$$

The sediment water content was estimated in section 2.1 of the materials and methods of the MS where the sediment collection and post-processing approach is described. Different masses of the sediment sample were weighed, dried, and then weighed again to determine the average ratio of the dry the wet sediment weight. This was determined to be $\frac{m_{dry}}{m_s} = 0.38$. Now, equation (S8) for aerobic bacteria reduces to:

$$\Delta\dot{T}_{sediment} = \frac{1.50 \times 10^{13} \times 0.38 \times \dot{Q}_{cell}}{c_s}$$

$$\Delta\dot{T}_{sediment} = \frac{4.58}{\mathrm{c_s}} \,°C/s \tag{S9}$$

For anaerobically grown *E. coli*, with Q_b = 0.20 pW, the equation becomes:

$$\Delta\dot{T}_{sediment} = \frac{1.15}{c_s} \,°C/s \tag{S10}$$

Discussion SD2: Estimating the Range of the Specific Heat Capacity

To calculate the expected specific heat capacity of the sediment used in this study, the approach of [43] was used. They incorporated the presence of dry sediment, water, organic matter, and air into their calculations. As the mass of dissolved air is negligible, and the organic matter content of the used sample is unknown, only the contributions of dry sediment and water were considered. Assuming the specific heat capacity of water is $c_w = 4180$ J/kg°C (between 25-30 °C)[45], and taking into account the dry sediment content of the used sediment (α_d = 0.38) one can estimate the specific heat capacity of the sediment used in this study with the following equation used by[43]:

$$(m_d + m_w)c_s = m_w c_w + m_d c_d$$

$$c_s = \frac{m_w c_w + m_d c_d}{m_d + m_w} \tag{S11}$$

Where m_d and m_w are the mass of water and dry sediment in the sample respectively, and c_d is the specific heat capacity of dry sediment, and as explained earlier, c_s and c_w are the specific heat capacities of the sediment sample and water respectively. To determine c_d,[44] performed a literature review, and used a calorimetric method to determine the heat capacity of 9 samples of dry sediment with different organic matter and tight-bound water content. They arrived at an average value that is close to that of literature, namely: c_d =750 ± 20 J/kg°C for the diverse samples.

Dividing the numerator and denominator of equation (S11) by the total sample mass m_s, and using $\alpha_d = \frac{m_d}{m_s} = 0.38$ and $\alpha_w = 1 - \alpha_d = 0.62$ one obtains:

$$c_s = \frac{\alpha_w c_w + \alpha_d c_d}{\alpha_w + \alpha_d} \tag{S12}$$

$$= \frac{0.62 \times 4180 \left[\frac{J}{kg°C}\right] + 0.38 \times 750 \left[\frac{J}{kg°C}\right]}{1}$$

The equation yields a value of c_s = 2880 J/kg°C, which closely approximates those already found in the literature (Table 1 in the MS). The heat capacity found in the experiment however is almost double the theoretically predicted value. To estimate the upper bound of the specific heat capacity, a variant of equation (S12) that accounts for water, soil particle, and organic matter

content is considered. As heat capacity of organic matter is c_o=1920 J/kg°C according to [55], the combined specific heat capacity would be given as:[43]

$$c_s = \alpha_w c_w + \alpha_d c_d + \alpha_o c_o \quad \text{(S13)}$$

Where $\alpha_o = \frac{m_o}{ms}$ is the mass ratio of organic matter to the entire sample, and c_o is the heat capacity of organic matter. Since this is a weighted sum equation, it must be that $\alpha_w + \alpha_d + \alpha_o = 1$, and that each of $\alpha_d, \alpha_w,$ and $\alpha_o \leq 1$. Equation (S13) then becomes:

$$c_s = 4180\alpha_w + 730\alpha_d + 1900\alpha_o$$

Under the given constraints ($\alpha_w + \alpha_d + \alpha_o = 1$, and each of $\alpha_d, \alpha_w, \alpha_o \leq 1$), this equation has an upper bound of 4180 J/kg°C, namely the specific heat capacity of water (Compare to Table S2, where a range of sediment specific heat capacity values are summarized for different soil types and their water content).

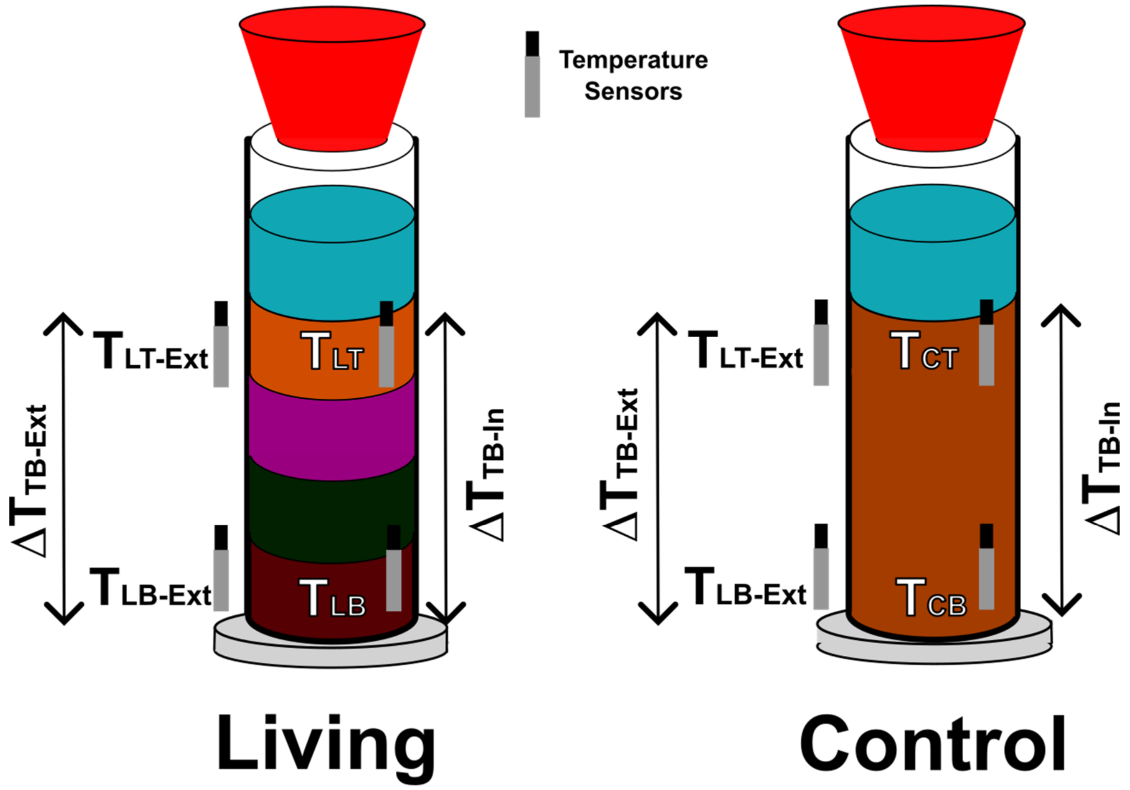

Figure S8. Measurement parameters used for vertical temperature comparison. Schematic representation of the parameters analyzed to evaluate temperature gradients with column height (related to Section 3.4). Internal temperatures at the living column top (T_{LT}) and bottom (T_{LB}) were recorded together with their external counterparts ($T_{LT\text{-}Ext}$, $T_{LB\text{-}Ext}$). The same configuration was applied to the control column (Abbreviations: T_{CT} = control top, T_{CB} = control bottom, $T_{CT\text{-}Ext}$, $T_{CB\text{-}Ext}$ for their external counterparts). For each column, temperature differences between the top and bottom positions were calculated for internal ($\Delta T_{TB\text{-}In}$) and external ($\Delta T_{TB\text{-}Ext}$) sensors to assess vertical thermal gradients.

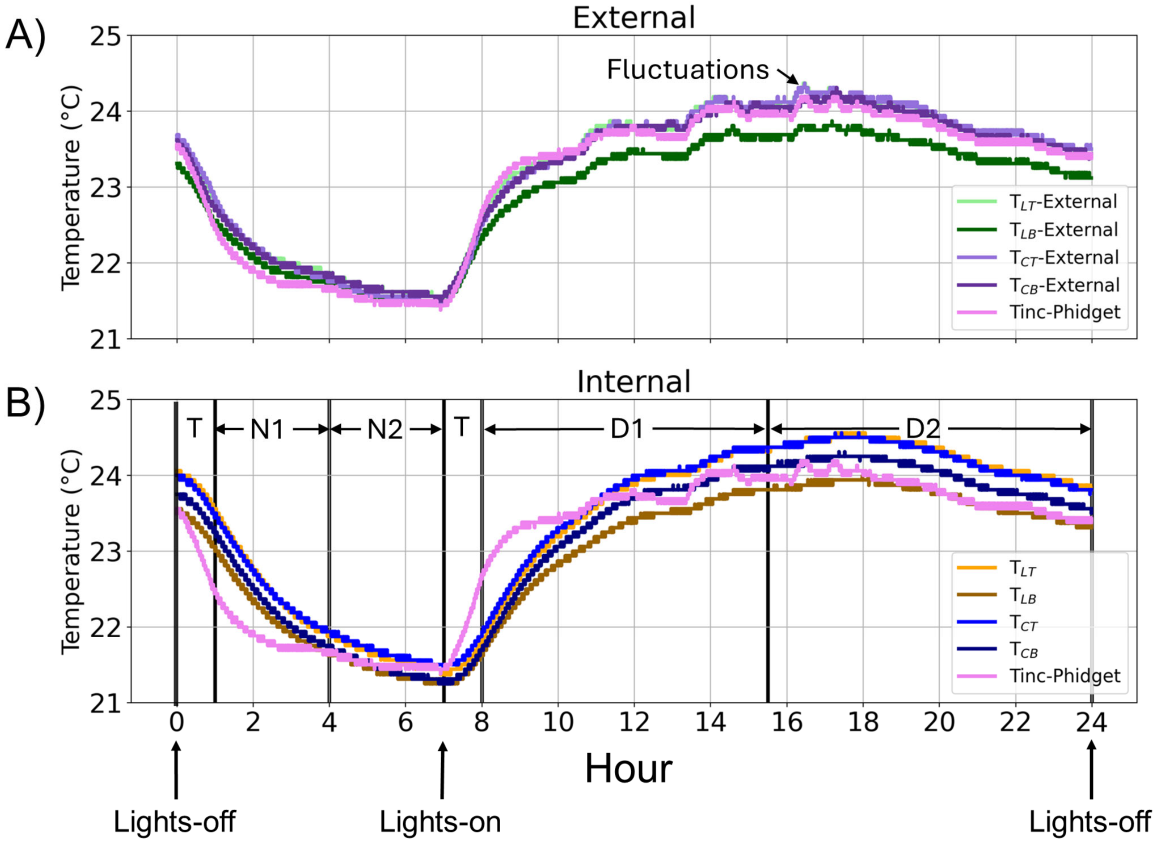

Figure S9. Comparison of external and internal temperature profiles and definition of illumination phases. A) Temperature profiles recorded by DS18B20 sensors placed outside the Winogradsky columns adjacent to the living top (T_{LT}-External, cyan), living bottom (T_{LB}-External, dark green), control top (T_{CT}-External, light purple), and control bottom (T_{CB}-External, dark purple) regions, along with the incubator reference temperature (T_{Inc}-Phidget, pink). No measurable lag is observed between external sensors and the incubator reference. B) Internal column temperatures recorded simultaneously, showing a persistent lag relative to incubator temperature changes, particularly during sharp transitions. The 24-h illumination cycle is divided into four quarters (N1, N2, D1, and D29 to facilitate calculation of average values while preserving dynamic effects across the cycle. Transitional phases (T1 = 00:00–01:00, T2 = 07:00–08:00) mark gradual light intensity changes simulating sunset and sunrise.

Discussion SD3 - Analysis of temporal lag in column temperature profiles

Temperature data acquired by external DS18B20 sensors revealed no measurable delay between the external sensor readings and those of the incubator reference (T_{Inc}, Phidget sensor) (Figure S9A, compare pink curve to other traces). This confirms that the temporal lag previously observed in internal column temperatures originates primarily from the thermal inertia of the sediment rather than from the sensors themselves. The apparent delay in the living-bottom temperature (Figure S9A, dark green) likely results from its deeper positioning, which caused slightly lower steady-state values compared with the top region.

Minor short-term oscillations detected by the external sensors (Figure S9A, arrows) were absent from the corresponding internal temperature profiles (Figure S9B). These small fluctuations likely reflect ambient variations within the open incubator—such as periodic switching of room lights or airflow—while the sediment and PMMA column walls dampened these effects internally through their heat capacity and insulation.

For subsequent quantitative analysis of vertical and temporal temperature gradients (Section 3.4), the 24-h illumination cycle (08:00–00:00 day, 01:00–07:00 night) was divided into four defined periods (Figure S9B): N1 (01:00–04:00), N2 (04:00–07:00), D1 (08:00–15:30), and D2 (15:30–00:00). The transitional phases corresponding to sunset (T1, 00:00–01:00) and sunrise (T2, 07:00–08:00) were excluded from averaging because of programmed lamp intensity changes.

These defined intervals were used to calculate mean temperature values and differences between the top and bottom regions for both living and control columns.

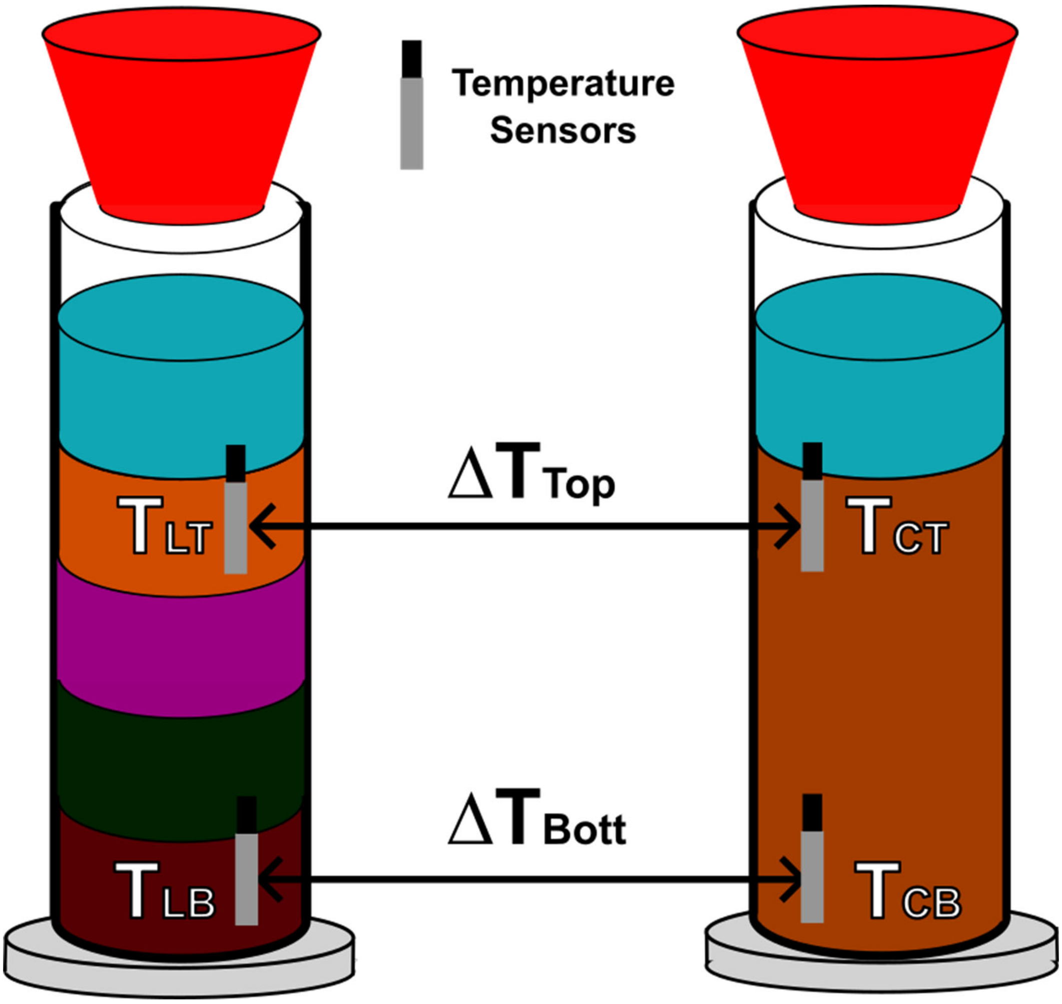

Figure S10. Illustration of the parameters used to investigate temperature differences over months (section 3.5). In order to search for patterns indicating microbial metabolic activity, focus was placed on searching for patterns in the temperature difference between the living and control column top regions (T_{LT} and T_{CT} respectively, where $\Delta T_{Top}=T_{LT}-T_{CT}$), as well as living and control bottom regions (T_{LB} and T_{CB} respectively, where $\Delta T_{Bott}=T_{LB}-T_{CB}$).

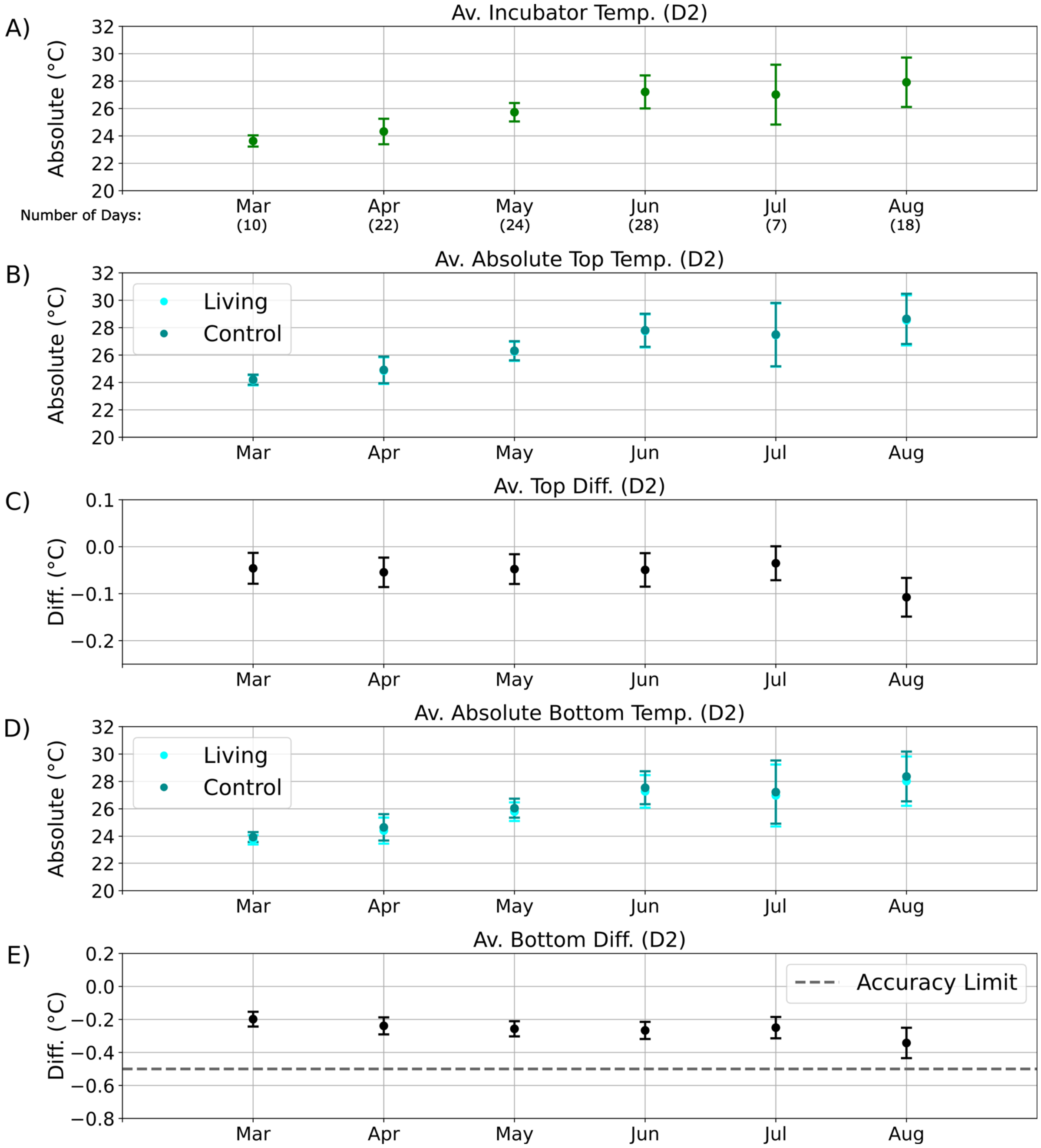

Figure S11. Long-term temperature analysis for the D2 phase of the day–night cycle. Average temperatures and temperature differences recorded over six months during the D2 illumination phase. The *x*-axis indicates the corresponding months of data collection, including the number of valid measurement days without technical interruptions. A) Mean incubator temperature. B) Average absolute temperature of the top regions of the living (cyan) and control (dark cyan) columns. C) Mean top temperature difference (ΔT_{Top}) between living and control columns. D) Average absolute temperature of the bottom regions of the living (cyan) and control (dark cyan) columns. E) Mean bottom temperature difference (ΔT_{Bott}) between living and control columns.

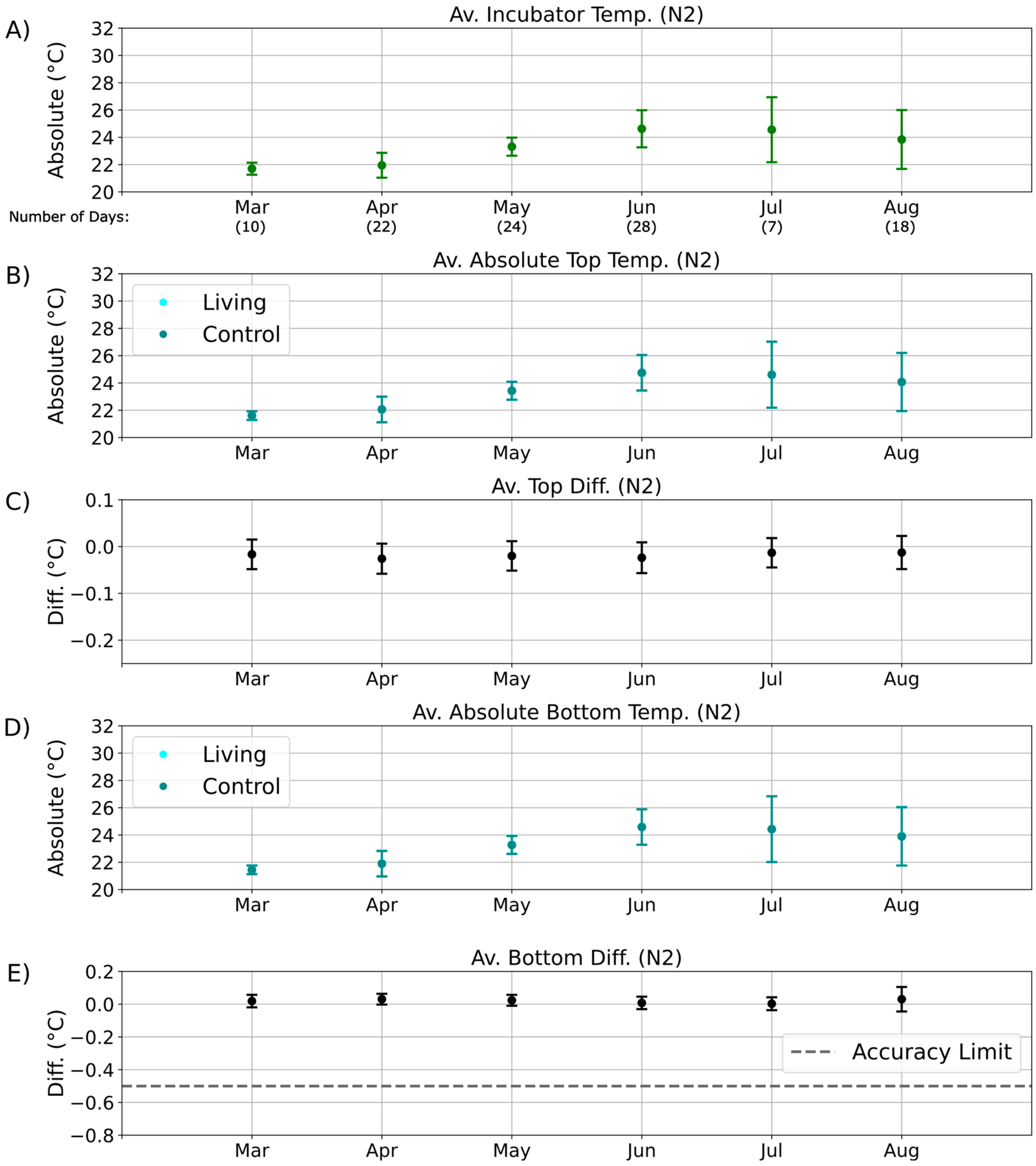

Figure S12. **Long-term temperature analysis for the N2 phase of the day–night cycle.** Average temperatures and temperature differences recorded over six months during the N2 nighttime phase. The x-axis indicates the corresponding months of data collection, including the number of valid measurement days without technical interruptions. A) Mean incubator temperature. B) Average absolute temperature of the top regions of the living (cyan) and control (dark cyan) columns; data points for the living column overlap with those of the control and are therefore not visible. C) Mean top temperature difference (ΔT_{Top}) between living and control columns. D) Average absolute temperature of the bottom regions of the living (cyan) and control (dark cyan) columns; data points for the living column overlap with those of the control and are therefore not visible. E) Mean bottom temperature difference (ΔT_{Bott}) between living and control columns.

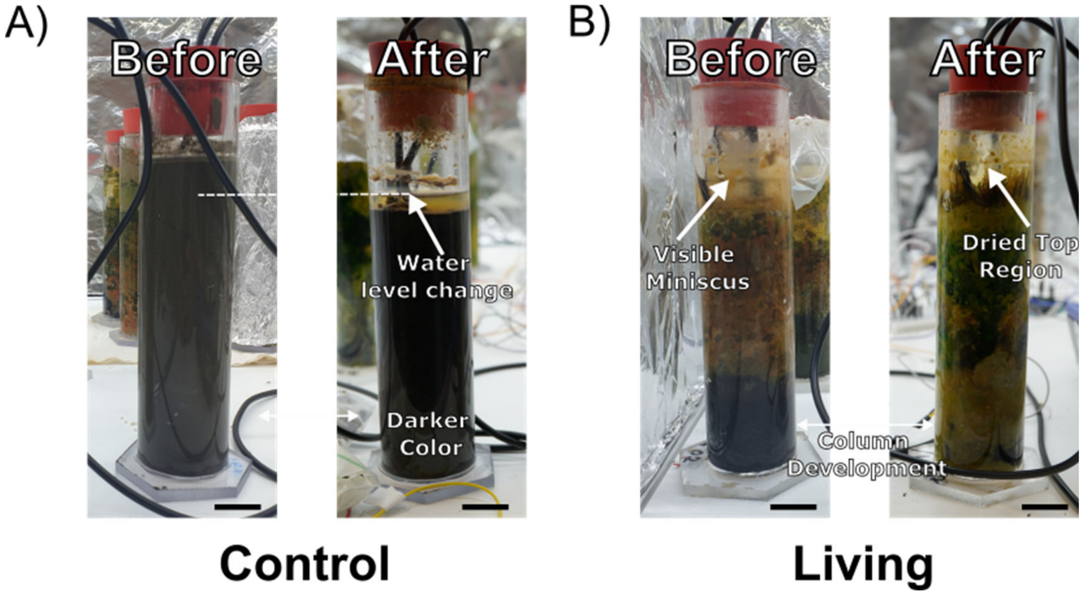

Figure S13. Physical changes in the Winogradsky columns during the course of the experiment. Photographs documenting visible alterations in the columns between the start of the experiment (November 2022, "Before") and two months after the August data point shown in previous figures (November 2024, "After"). A) Control column before and after darkening of the sediment, showing both sediment color change and partial evaporation of the supernatant in the upper region. B) Living column before and after supernatant evaporation, revealing additional layer development and structural changes in the sediment. Scale bars are 20 mm for all images.

Discussion SD4: Evidence of microbial growth in the control column

Over the course of the experiment, the control column exhibited a gradual darkening of the sediment (Figure S13A), suggesting the possible onset of biological activity, most likely associated with the growth of sulfate-reducing bacteria. This observation indicates that the initially abiotic control column may have transitioned to a biotic state. The most plausible explanation is a slow ingress of air through the rubber stopper, which, although nominally airtight, could have allowed minimal oxygen leakage and subsequent microbial colonization.

In parallel, the living column showed marked evaporation of the supernatant (Figure S13B). The reduction in surface water likely diminished photosynthetic activity by Cyanobacteriota and algae, thereby disrupting the oxygen gradient and associated nutrient cycling within the column. Such changes could have altered the internal microbial community composition and reduced metabolic heat generation.

Together, these processes provide a possible explanation for the apparent emergence of metabolic activity in the control column during later stages of the experiment. The effect was predominantly observed under illuminated conditions, while nighttime data showed no such differences, suggesting that the unintended microbial colonization of the control column was photoresponsive or more active in the presence of light.

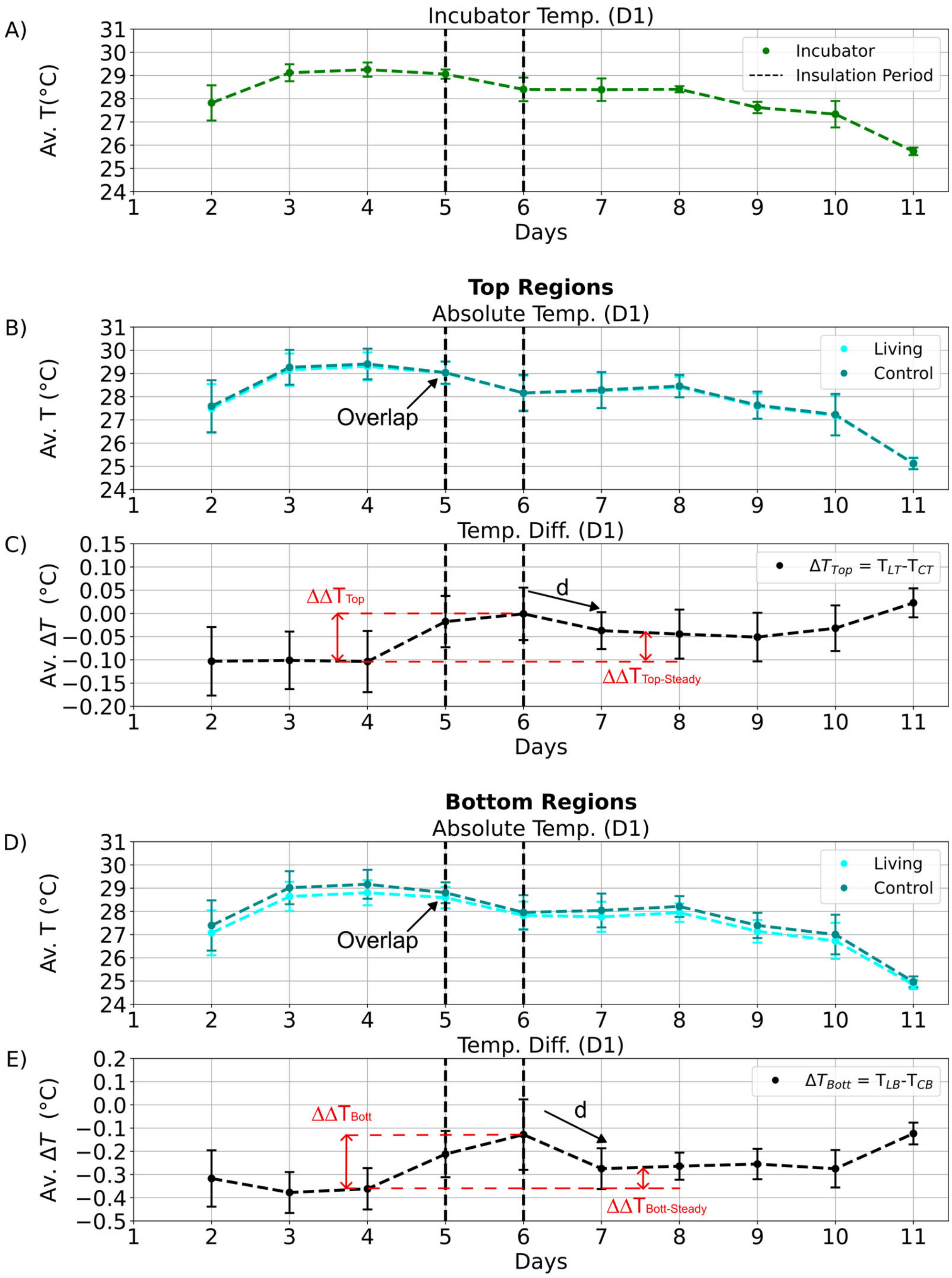

Figure S14. Daytime temperature dynamics during the aluminum foil insulation experiment (D1 phase). Average temperatures and temperature differences between the living and control Winogradsky columns during the D1 quarter of the day–night cycle. Error bars represent standard deviations, and black dashed lines mark the start and end of the insulation period. A) Mean incubator temperature. B) Average absolute temperature of the top regions of the living (cyan) and control (dark cyan) columns. C) Average temperature difference between the top regions of the living and control columns. Red dashed lines indicate the calculated temperature retention ($\Delta\Delta T_{Top}$ and $\Delta\Delta T_{Bott}$) and steady-state temperature difference ($\Delta\Delta T_{Top\text{-}Steady}$ and $\Delta\Delta T_{Bott\text{-}Steady}$), with arrows marking the decline from maximum values. D) Average absolute temperature of the bottom regions of the living (cyan) and control (dark cyan) columns. E) Average temperature difference between the bottom regions of the living and control columns.

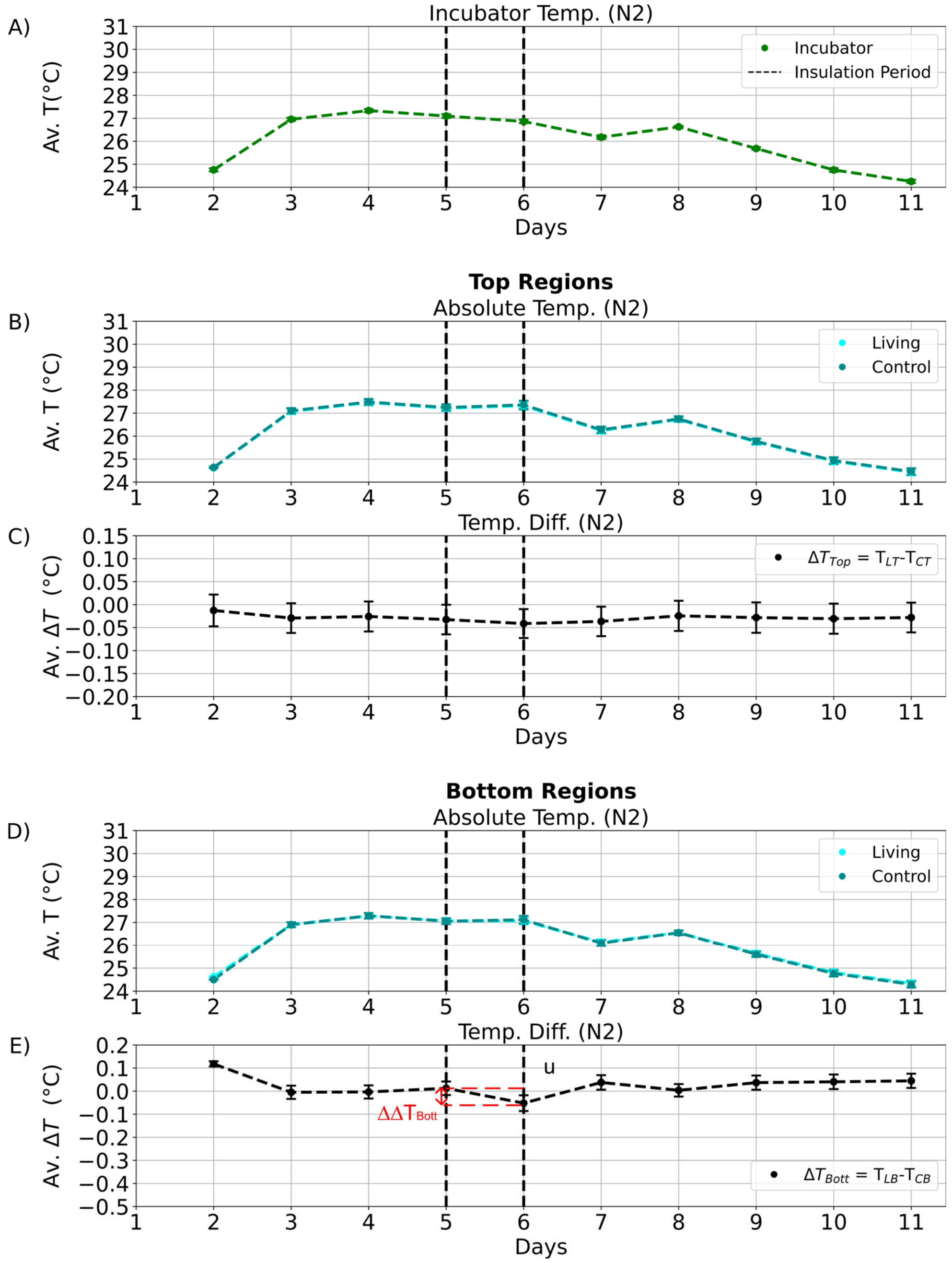

Figure S15. Nighttime temperature dynamics during the aluminum foil insulation experiment (N2 phase). Average temperatures and temperature differences between the living and control Winogradsky columns during the N2 quarter of the day–night cycle. Error bars represent standard deviations, and black dashed lines mark the start and end of the insulation period. A) Mean incubator temperature. B) Average absolute temperature of the top regions of the living (cyan) and control (dark cyan) columns. C) Average temperature difference between the top regions of the living and control columns. Red dashed lines indicate the calculated temperature retention ($\Delta\Delta T_{Top}$ and $\Delta\Delta T_{Bott}$) and steady-state temperature difference ($\Delta\Delta T_{Top\text{-}Steady}$ and $\Delta\Delta T_{Bott\text{-}Steady}$), with arrows marking the decline from maximum values. D) Average absolute temperature of the bottom regions of the living (cyan) and control (dark cyan) columns. E) Average temperature difference between the bottom regions of the living and control columns.

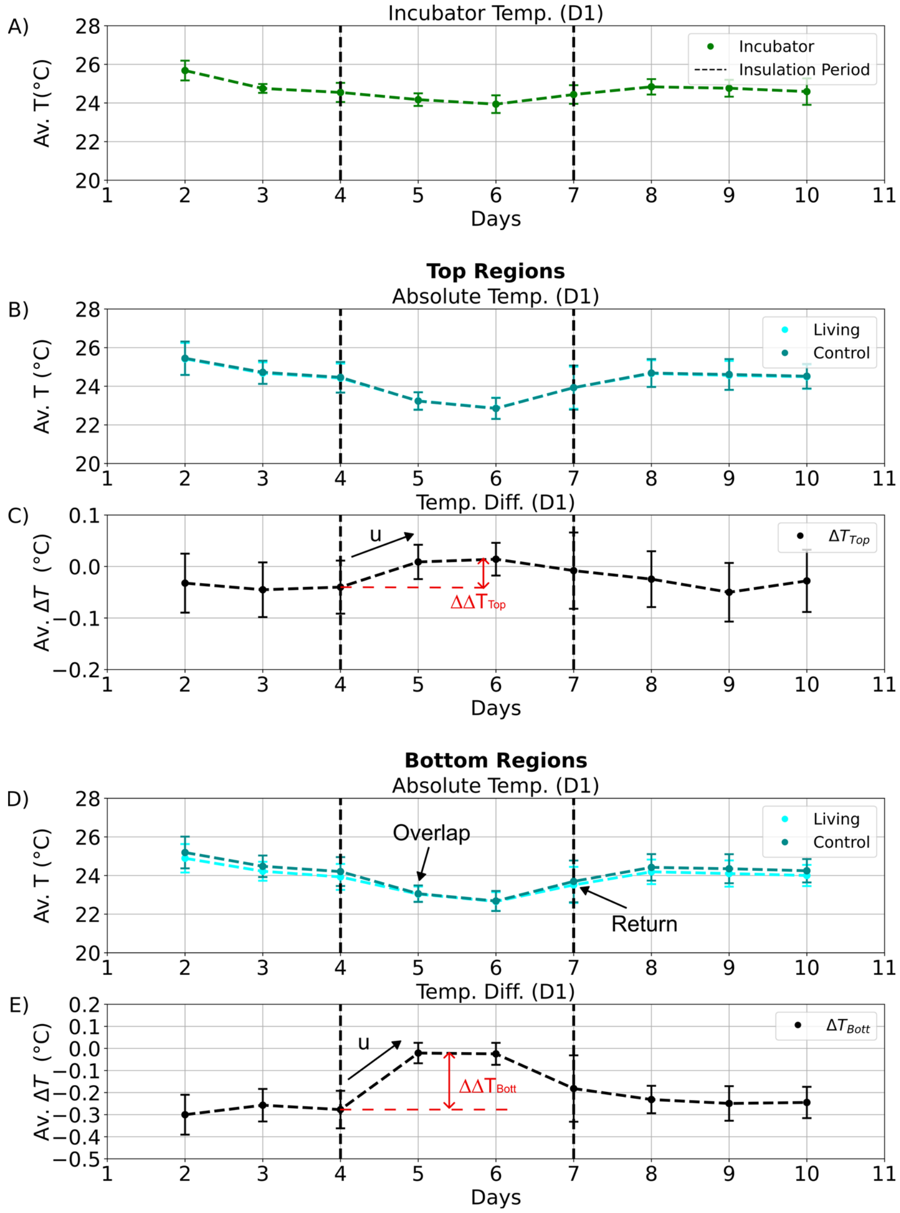

Figure S16. **Daytime temperature dynamics during the second aluminum foil insulation experiment (D1 phase).** Average temperatures and temperature differences between the living and control Winogradsky columns during the D1 quarter of the day–night cycle. Error bars represent standard deviations, and dashed vertical lines indicate the start and end of the insulation period. A) Mean incubator temperature. B) Average absolute temperatures of the top regions of the living (cyan) and control (dark cyan) columns. C) Average temperature difference between the living and control top regions. D) Average absolute temperatures of the bottom regions of the living and control columns. E) Average temperature difference between the bottom regions of the living and control columns over several days. Arrows highlight temperature changes in the living column relative to the control as a result of insulation. Red dashed lines denote the calculated temperature retention ($\Delta\Delta T_{Top}$ and $\Delta\Delta T_{Bott}$) and steady-state temperature differences ($\Delta\Delta T_{Top\text{-}Steady}$ and $\Delta\Delta T_{Bott\text{-}Steady}$), with arrows indicating notable changes.

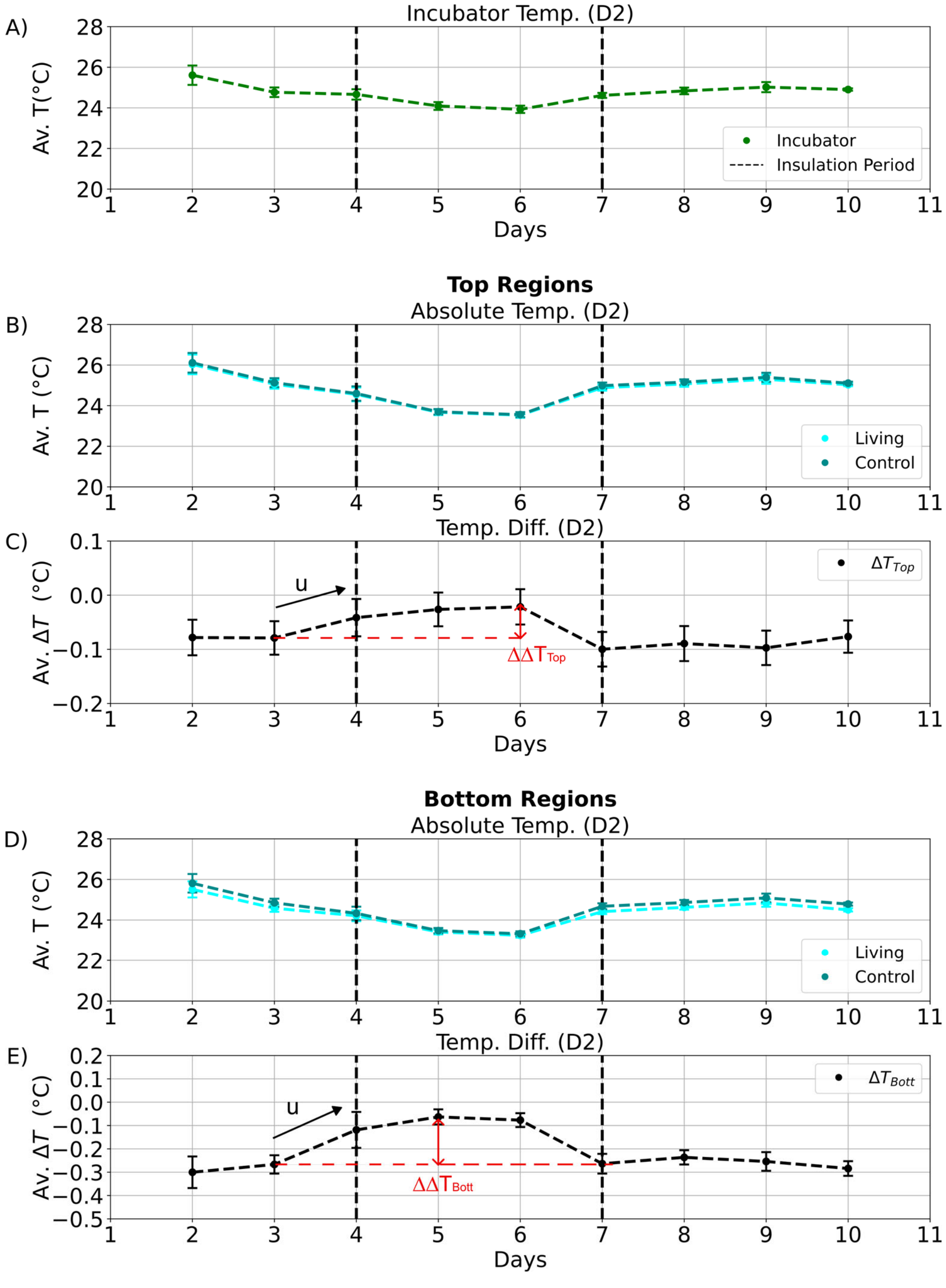

Figure S17. Daytime temperature dynamics during the second aluminum foil insulation experiment (D2 phase). Average temperatures and temperature differences between the living and control Winogradsky columns during the D2 quarter of the day–night cycle. Error bars represent standard deviations, and dashed vertical lines mark the start and end of the insulation period. A) Mean incubator temperature. B) Average absolute temperatures of the top regions of the living (cyan) and control (dark cyan) columns. C) Average temperature difference between the living and control top regions. D) Average absolute temperatures of the bottom regions of the living and control columns. E) Average temperature difference between the bottom regions of the living and control columns over several days. Arrows highlight temperature changes in the living column relative to the control observed during insulation.

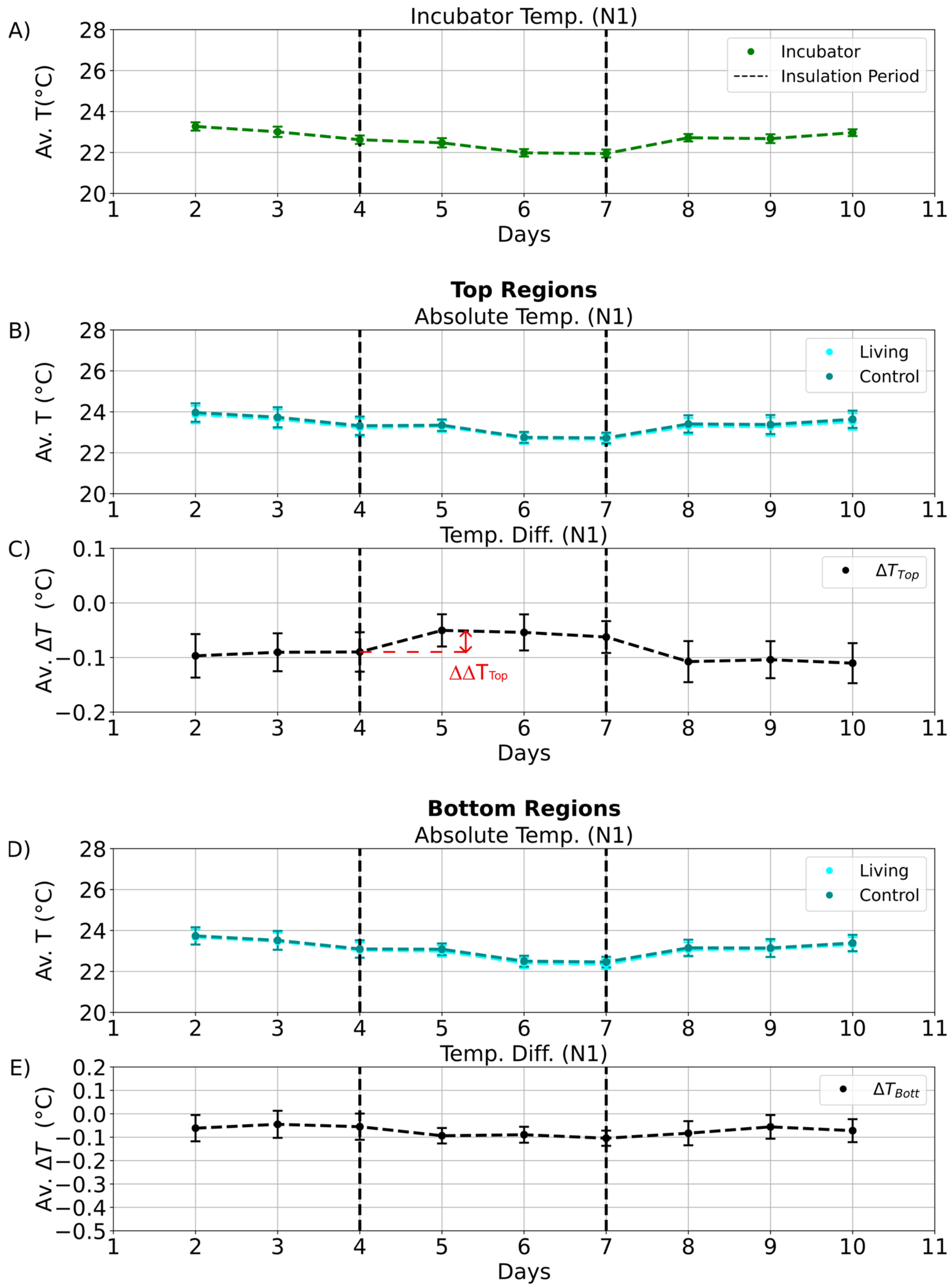

Figure S18. Nighttime temperature dynamics during the second aluminum foil insulation experiment (N1 phase). Average temperatures and temperature differences between the living and control Winogradsky columns during the N1 quarter of the day–night cycle. Error bars represent standard deviations, and dashed vertical lines mark the start and end of the insulation period. A) Mean incubator temperature. B) Average absolute temperatures of the top regions of the living (cyan) and control (dark cyan) columns. C) Average temperature difference between the living and control top regions. D) Average absolute temperatures of the bottom regions of the living (cyan) and control (dark cyan) columns. E) Average temperature difference between the bottom regions of the living and control columns over several days. Arrows highlight temperature changes in the living column relative to the control observed during insulation.

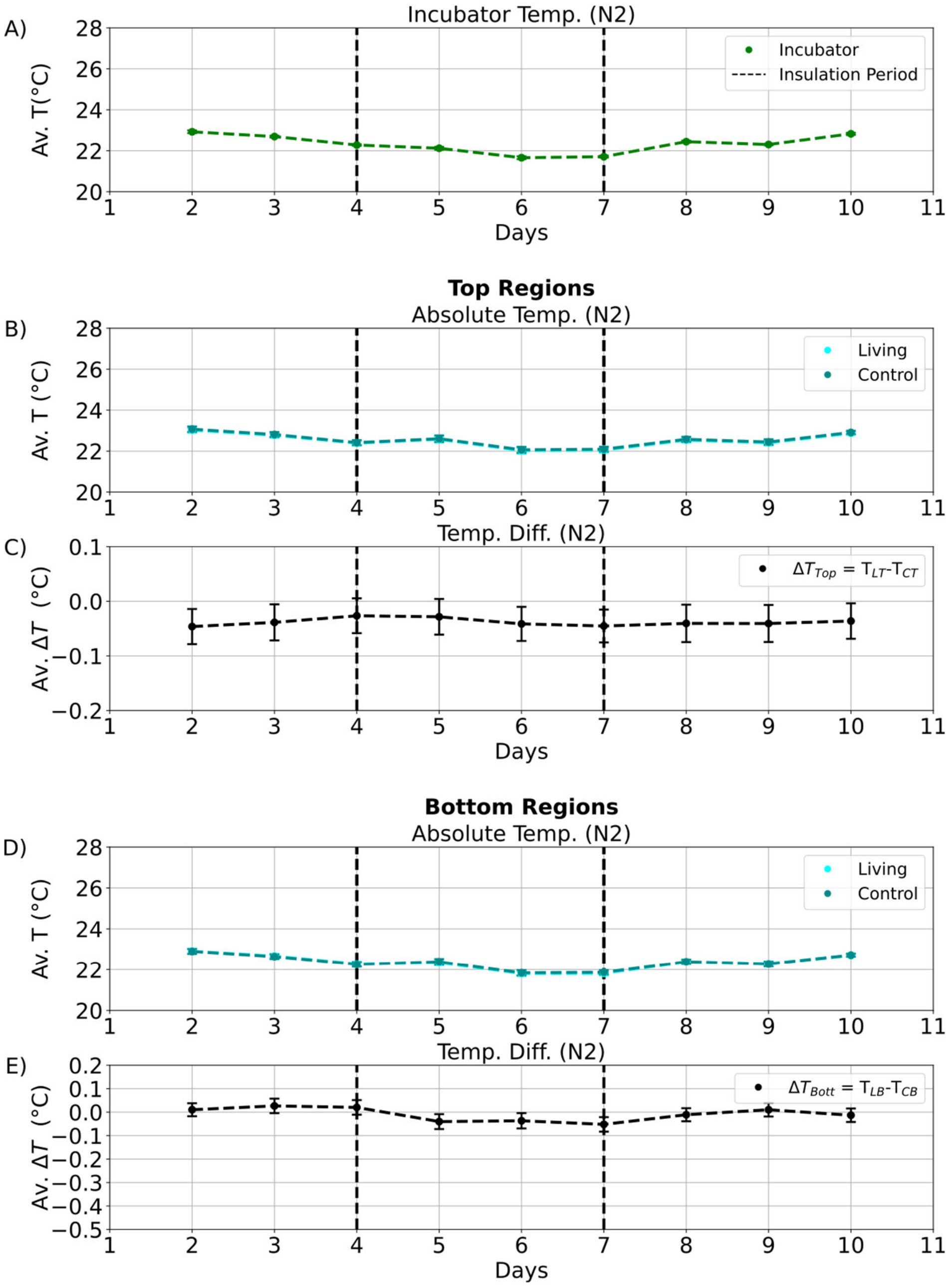

Figure S19. Nighttime temperature dynamics during the second aluminum foil insulation experiment (N2 phase). Average temperatures and temperature differences between the living and control Winogradsky columns during the N2 quarter of the day–night cycle. Error bars represent standard deviations, and dashed vertical lines mark the start and end of the insulation period. A) Mean incubator temperature. B) Average absolute temperatures of the top regions of the living (cyan) and control (dark cyan) columns. C) Average temperature difference between the living (cyan) and control (dark cyan) top regions. D) Average absolute temperatures of the bottom regions of the living and control columns. E) Average temperature difference between the bottom regions of the living and control columns over several days. Arrows highlight temperature changes in the living column relative to the control observed during insulation.

Discussion SD5 – Analysis of temperature changes during the extended (4-day) insulation experiment

This discussion provides a detailed evaluation of the second insulation experiment, in which the Winogradsky columns were unhydrated and wrapped in aluminum foil for an extended period of four days. During the D1 phase, insulation enabled the living column to retain heat more effectively than the control, particularly in the bottom region. This is reflected by overlapping temperature profiles in the lower part of the columns (Figure S16D, “Overlap”) and by the increase in temperature difference between the living and control columns (Figures S16C and S16E; $\Delta\Delta T_{Top}$ and $\Delta\Delta T_{Bott}$). The effect likely resulted from a reduction in phototrophic activity in the upper layers deprived of light, while dark-adapted microorganisms in the lower regions remained metabolically active. After insulation was removed, the absolute temperature relationships reverted to their previous state (Figures S16C, S16E, and S16D “Return”), indicating that the effect was transient and primarily dependent on the duration of insulation.

Similar patterns were observed during the D2 phase (Figures S17C and S17E), where the living column again retained slightly more heat than the control. These findings suggest that microbial activity in the sediment partially compensated for the absence of external illumination. However, the prolonged lack of light likely disturbed the balance of the microbial communities, preventing the sustained elevation of temperature observed after shorter insulation periods.

During the nighttime phases, the effect was weaker and primarily evident in the upper regions during N1 (Figure S18C, $\Delta\Delta T_{Top}$). This pattern suggests gradual heat loss from the living column following decreases in incubator temperature, with residual heat dissipating slowly from the lower sediment layers. The base of the column was not covered by foil, facilitating heat dissipation and explaining the absence of significant retention or temperature variation at the bottom during N1 and N2 (Figures S18–S19E, where the black curve remains nearly constant).

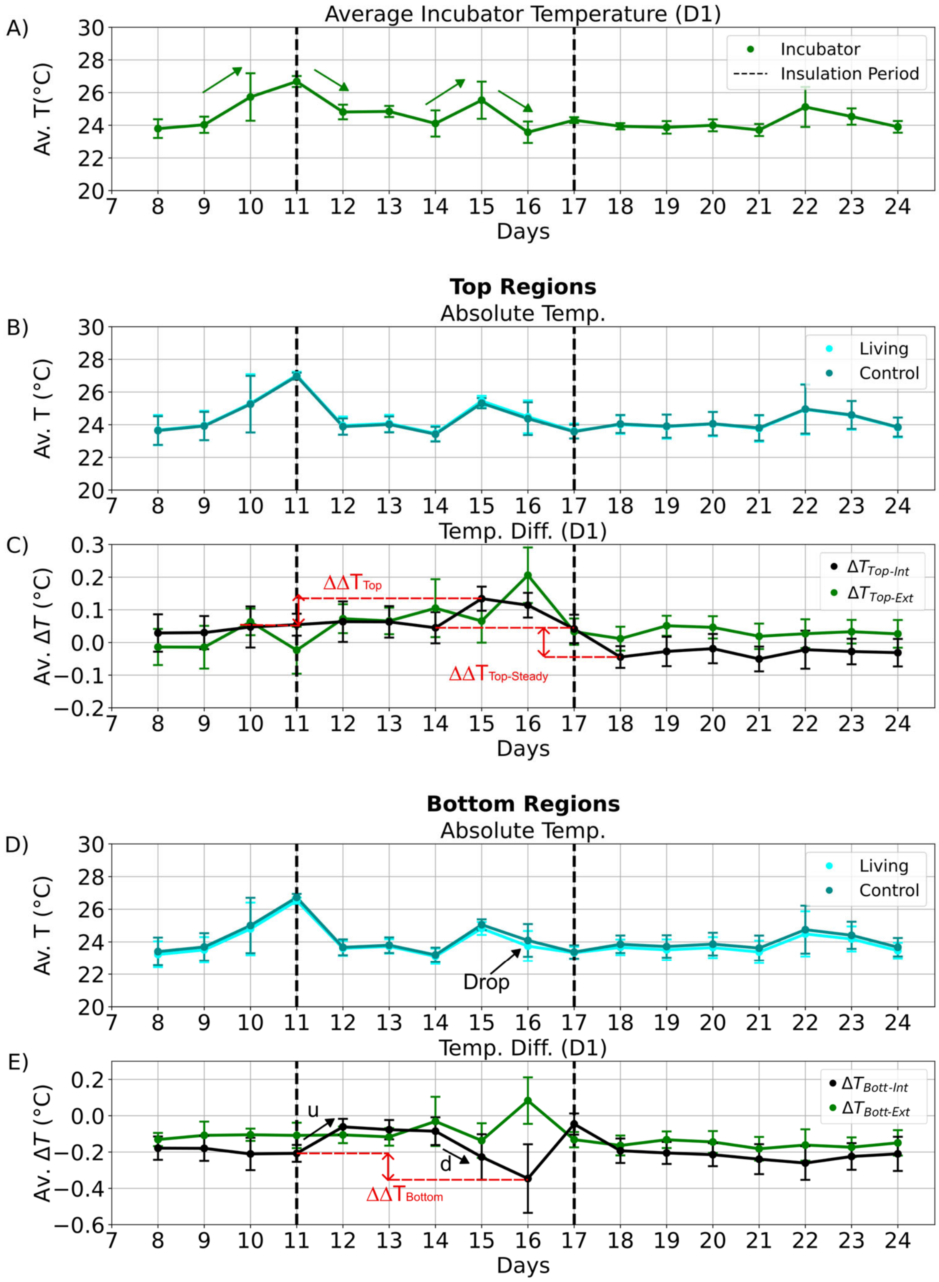

Figure S20. Daytime temperature dynamics during the third insulation experiment (D1 phase). Average temperatures and temperature differences between the living and control Winogradsky columns after insulation with polyethylene (PE) and aluminum foil. A) Mean incubator temperature. B) Average absolute temperatures of the top regions of the living (cyan) and control (dark cyan) columns. C) Internal (black) and external (green) average temperature differences between the top regions of the living and control columns (Abbreviations: respectively $\Delta T_{Top\text{-}Int}$ and $\Delta T_{Top\text{-}Ext}$). D) Average absolute temperatures of the bottom regions of the living (cyan) and control (dark cyan) columns. E) Internal (black) and external (green) average temperature differences between the bottom regions of the living and control columns (Abbreviations: respectively $\Delta T_{Bott\text{-}Int}$ and $\Delta T_{Bott\text{-}Ext}$). Error bars represent standard deviations, and black dashed lines mark the start and end of the insulation period. Arrows highlight key temperature change events, and the shaded region indicates the interval during which the columns were insulated with the Al–PE foil.

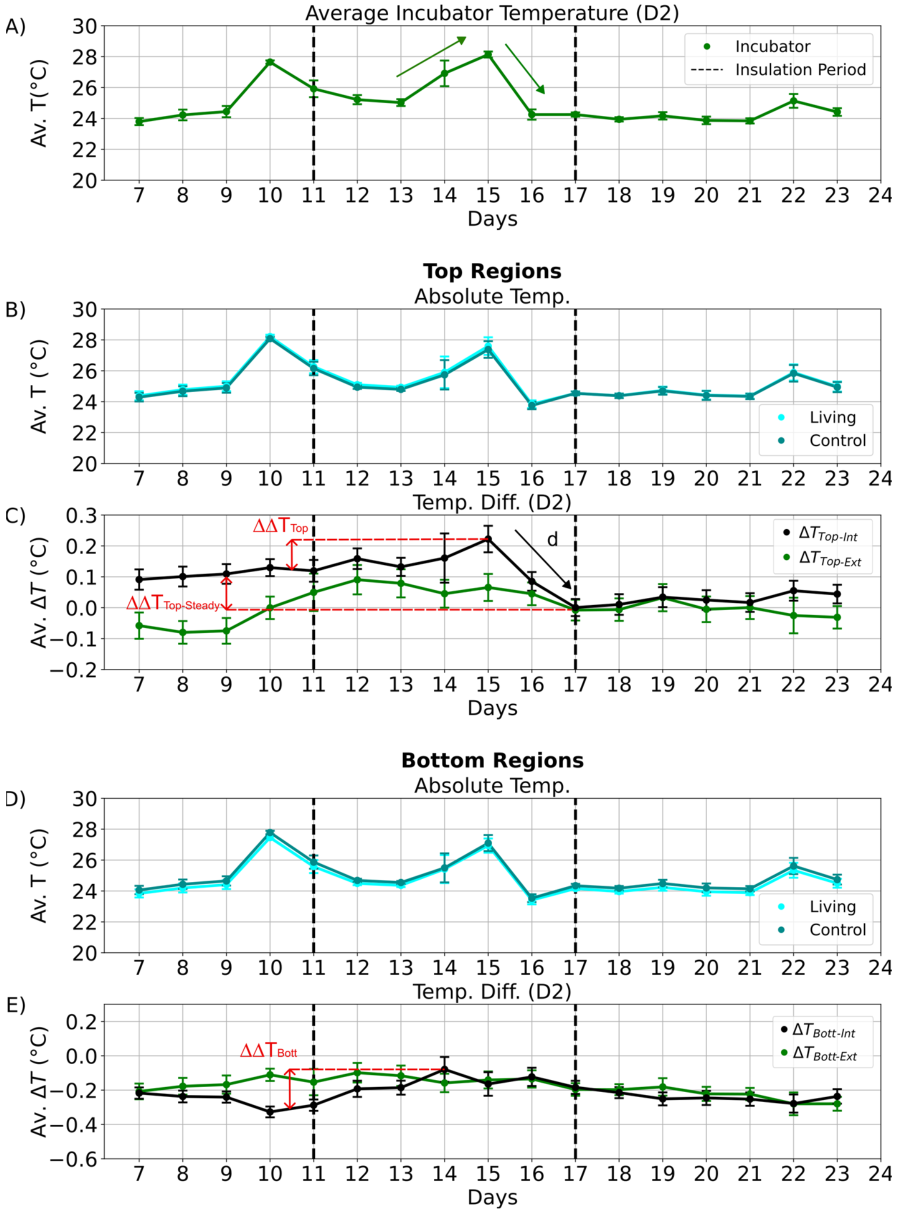

Figure S21. **Daytime temperature dynamics during the third insulation experiment (D2 phase).** Average temperatures and temperature differences between the living and control Winogradsky columns after insulation with polyethylene (PE) and aluminum foil. A) Mean incubator temperature. B) Average absolute temperatures of the top regions of the living (cyan) and control (dark cyan) columns. C) Internal (black) and external (green) average temperature differences between the top regions of the living and control columns (Abbreviations: respectively $\Delta T_{Top\text{-}Int}$ and $\Delta T_{Top\text{-}Ext}$). D) Average absolute temperatures of the bottom regions of the living (cyan) and control (dark cyan) columns. E) Internal (black) and external (green) average temperature differences between the bottom regions of the living and control columns (Abbreviations: respectively $\Delta T_{Bott\text{-}Int}$ and $\Delta T_{Bott\text{-}Ext}$). Error bars represent standard deviations, and black dashed lines mark the start and end of the insulation period. Arrows highlight key temperature change events, and the shaded region indicates the interval during which the columns were insulated with the Al–PE foil.

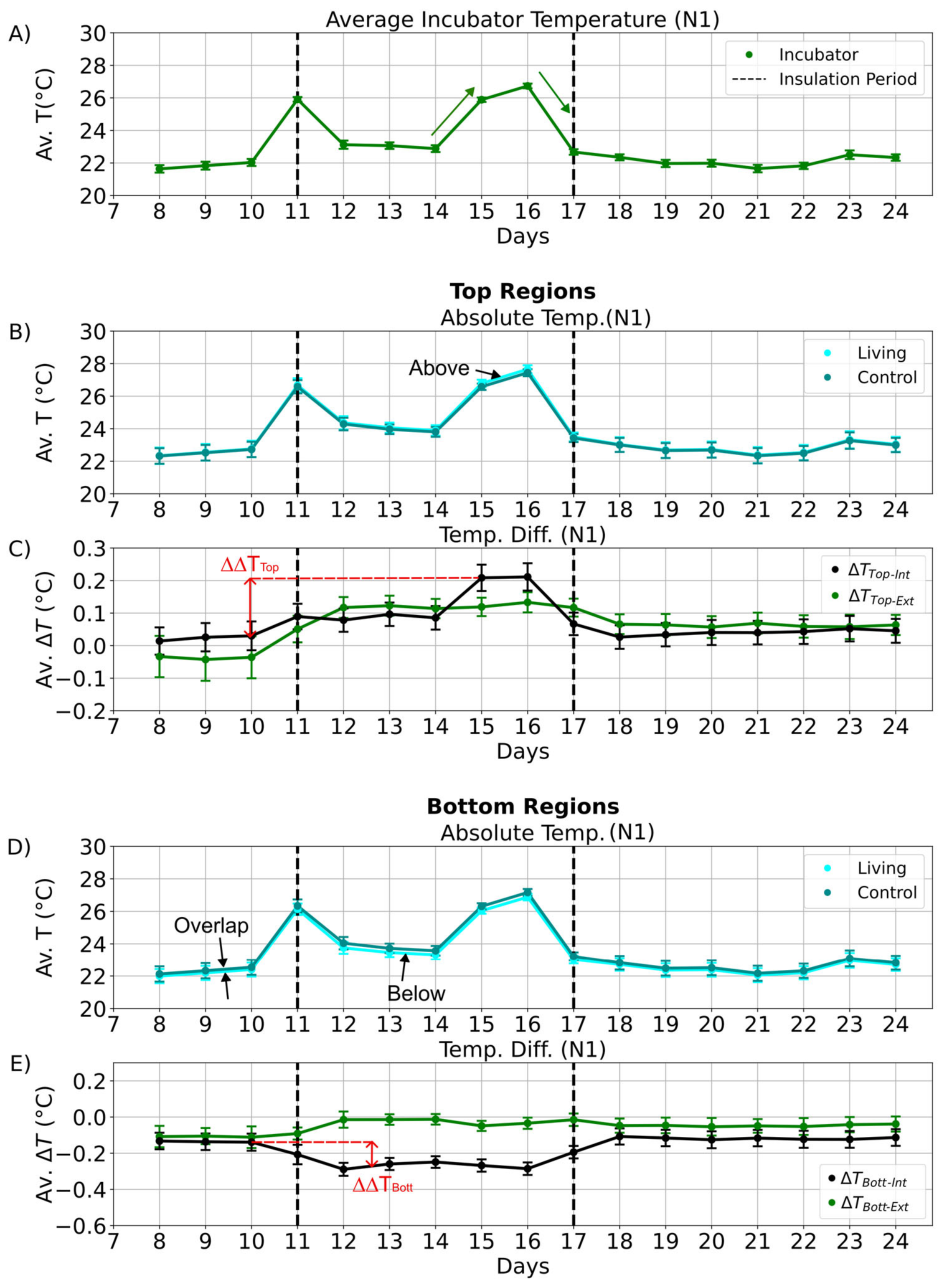

Figure S22. Nighttime temperature dynamics during the third insulation experiment (N1 phase). Average temperatures and temperature differences between the living and control Winogradsky columns after insulation with polyethylene (PE) and aluminum foil. A) Mean incubator temperature. B) Average absolute temperatures of the top regions of the living (cyan) and control (dark cyan) columns. C) Internal (black) and external (green) average temperature differences between the top regions of the living and control columns (Abbreviations: respectively $\Delta T_{Top\text{-}Int}$ and $\Delta T_{Top\text{-}Ext}$). D) Average absolute temperatures of the bottom regions of the living (cyan) and control (dark cyan) columns. E) Internal (black) and external (green) average temperature differences between the bottom regions of the living and control columns (Abbreviations: respectively $\Delta T_{Bott\text{-}Int}$ and $\Delta T_{Bott\text{-}Ext}$). Error bars represent standard deviations, and black dashed lines mark the start and end of the insulation period. Arrows highlight key temperature change events, and the shaded region indicates the interval during which the columns were insulated with the Al–PE foil.

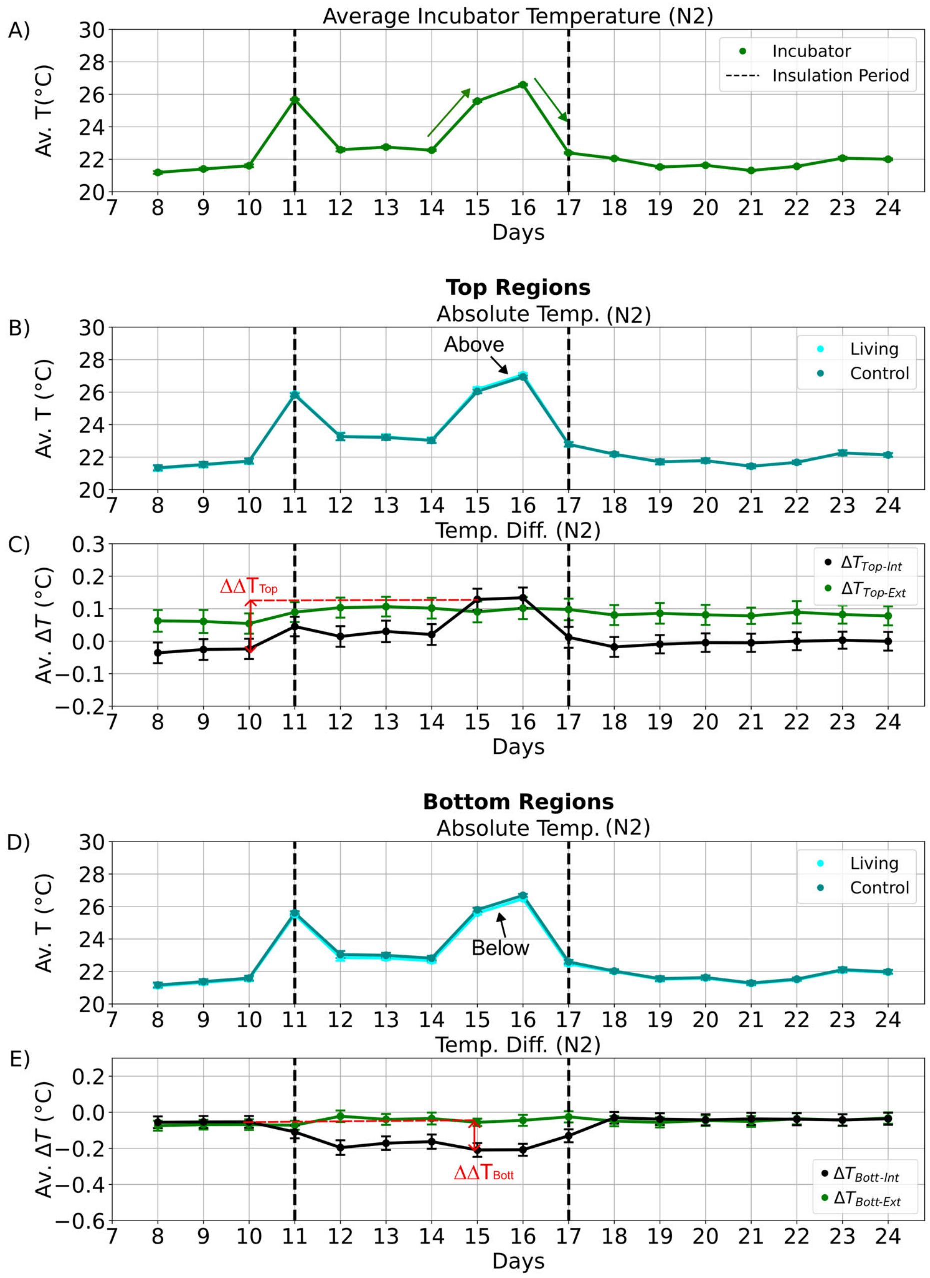

Figure S23. Nighttime temperature dynamics during the third insulation experiment (N2 phase). Average temperatures and temperature differences between the living and control Winogradsky columns after insulation with polyethylene (PE) and aluminum foil. A) Mean incubator temperature. B) Average absolute temperatures of the top regions of the living (cyan) and control (dark cyan) columns. C) Internal (black) and external (green) average temperature differences between the top regions of the living and control columns (Abbreviations: respectively $\Delta T_{Top\text{-}Int}$ and $\Delta T_{Top\text{-}Ext}$). D) Average absolute temperatures of the bottom regions of the living (cyan) and control (dark cyan) columns. E) Internal (black) and external (green) average temperature differences between the bottom regions of the living and control columns (Abbreviations: respectively $\Delta T_{Bott\text{-}Int}$ and $\Delta T_{Bott\text{-}Ext}$). Error bars represent standard deviations, and black dashed lines mark the start and end of the insulation period. Arrows highlight key temperature change events, and the shaded region indicates the interval during which the columns were insulated with the Al–PE foil.

Discussion SD6. Analysis of temperature changes during the Al–PE insulation experiment

This section provides a detailed analysis of the third insulation experiment, in which the rehydrated Winogradsky columns were wrapped in combined aluminum–polyethylene (Al–PE) insulation. During the experiment, the incubator temperature fluctuated substantially (green arrows in subfigures A in Figures S20-S23). External temperature traces were highly similar to and the incubator profile, confirming the overall stability of the surrounding thermal environment. To quantify external effects, additional temperature differences were calculated as $\Delta T_{Top-Ext}$ and $\Delta T_{Bott-Ext}$, representing the readings from the sensors positioned adjacent to the living and control columns (green curves in subfigures C, E in Figures S20-S23).

In the upper regions, internal temperature differences closely followed the external ones, indicating little deviation from the surrounding conditions (Figure S20C). However, after the insulation phase, the internal top temperature difference stabilized at a lower level than before (Figure S20C, $\Delta\Delta T_{Top-Steady}$, see black curve below green after the dashed interval), suggesting a shift in the surface microbial community due to prolonged darkness.

In the lower regions, the living column initially retained more heat than the control (Figure S20E, "u" arrow), but this advantage diminished toward the end of the insulation period (Figure S20E, "d" arrow). The living column's bottom temperature eventually fell below that of the control (Figure S20D, "Drop"), and the internal temperature differences diverged from the external gradients (Figure S20E, black vs. green curves), indicating that the observed effect was not driven by external incubator conditions. These findings imply that microbial metabolism initially maintained internal heat but gradually declined after several days without light, as reduced growth and lower metabolic rates resulted in diminished heat production

A similar trend was observed during the D2 phase (Figures S21C and S21E), where the internal top temperature difference ($\Delta\Delta T_{Top}$) decreased steadily ($\Delta\Delta T_{Top-Steady}$), suggesting that phototrophic microorganisms near the surface persisted temporarily by switching to alternative metabolic pathways before dying off under prolonged dark conditions. The sharp drop in top-region temperature differences coincided with a decrease in incubator temperature (Figure S21A, green arrows coincide with S21C "d" arrow, indicating that part of the observed signal may have been externally induced.

During the nighttime phases, the living column consistently exhibited slightly higher temperatures in the top region than the control, most pronounced by the latter half of the insulation phase (Figures S22B and S23B, see "Above" arrow indicating cyan curve above dark cyan). This pattern, also reflected in the increasing internal temperature differences (Figures S22C and S23C, $\Delta\Delta T_{Top}$), may indicate the activity of microbial populations entrained to the dark cycle. Although the existence of circadian rhythms in bacteria remains under debate [57], the repeated nighttime temperature increases suggest possible entrainment to the day–night cycle, as previously observed in *Cyanobacteriota* by Golden et al. [56]. In contrast, the bottom regions of the living column showed reduced heat retention (Figures S22E and S23E, $\Delta\Delta T_{Bott}$), a result that may be explained by microbial competition and stratified metabolic activity.

As noted earlier, the control column had shown signs of sulfate-reducing bacterial growth. Under extended dark conditions, these anaerobes may have become more active, producing H_2S and gas bubbles that promoted convective mixing and molecular transport. Such mixing could have redistributed heat and nutrients, favoring activity in the upper regions while suppressing anaerobic processes in the lower parts of the living column. However, this pattern may also have been influenced by external incubator fluctuations, since changes in incubator temperature (Figures S20–S23, subfigures A in all, green arrows) correspond closely with those in the upper-column temperature differences. Minor ambient fluctuations, such as periodic switching of laboratory lights, may also have contributed to small perturbations observed in the external sensor readings.

Although the observed variations were within the ±0.5 °C accuracy range of the DS18B20 sensors, they represent consistent trends recorded by the same probes and thus exceed random measurement noise. Sensor placement may nonetheless have affected absolute values, since full insulation shielded the sensors from radiant heat originating near the light source.

References

41. Maskow, T.; Paufler, S. What does calorimetry and thermodynamics of living cells tell us? *Methods* **2015**, *76*, 3-10, doi: https://doi.org/10.1016/j.ymeth.2014.10.035.
42. Torsvik, V.; Goksoyr, J.; Daae, F.L. High diversity in DNA of soil bacteria. *Appl. Environ. Microbiol.* **1990**, *56*, 782-787, doi: https://doi.org/10.1128/aem.56.3.782-787.1990.
43. Abu-Hamdeh, N.H. Thermal Properties of Soils as affected by Density and Water Content. *Biosyst. Eng.* **2003**, *86*, 97-102, doi: https://doi.org/10.1016/s1537-5110(03)00112-0
44. Wang, Y.; Lu, Y.; Horton, R.; Ren, T. Specific Heat Capacity of Soil Solids: Influences of Clay Content, Organic Matter, and Tightly Bound Water. *Soil Sci. Soc. Am. J.* **2019**, *83*, 1062-1066, doi: https://doi.org/10.2136/sssaj2018.11.0434.
45. National Insitute of Standards and Technology Water. Available online: https://webbook.nist.gov/cgi/cbook.cgi?ID=C7732185&Type=JANAFL&Plot=on (accessed on October 10, 2025).
47. Analog Devices *DS18B20: Programmable Resolution 1-Wire Digital Thermometer*; Analog Devices, Inc.: 2015.
51. Zhu, X.; Gao, Z.; Chen, T.; Wang, W.; Lu, C.; Zhang, Q. Study on the Thermophysical Properties and Influencing Factors of Regional Surface Shallow Rock and Soil in China. *Front. Earth Sci.* **2022**, *10*, doi: https://doi.org/10.3389/feart.2022.864548.
52. Goto, S.; Yamano, M.; Morita, S.; Kanamatsu, T.; Hachikubo, A.; Kataoka, S.; Tanahashi, M.; Matsumoto, R. Physical and thermal properties of mud-dominant sediment from the Joetsu Basin in the eastern margin of the Japan Sea. *Mar. Geophys. Res.* **2017**, *38*, 393-407, doi: https://doi.org/10.1007/s11001-017-9302-y.
53. Ghuman, B.S.; Lal, R. Thermal Conductivity, Thermal Diffusivity, and Thermal Capcity of Some Nigerian Soils. *Soil Sci.* **1985**, *139*, 74-80.
54. Yadav, M.; Saxena, G. Effect of compaction and moisture content on specific heat and thermal capacity of soils. *J. Indian Soc. Soil Sci.* **1973**, *21*, 129-132, doi.
55. Vries, D.A.; Wijk, W.R. Physics of Plant Environment. In *Thermal properties of soils*; Amsterdam, Netherlands: 1963.
56. Golden, S.S. Timekeeping in bacteria: the cyanobacterial circadian clock. *Curr. Opin. Microbiol.* **2003**, *6*, 535-540, doi: https://doi.org/10.1016/j.mib.2003.10.012.
57. Sartor, F.; Xu, X.; Popp, T.; Dodd, A.N.; Kovacs, A.T.; Merrow, M. The circadian clock of the bacterium B. subtilis evokes properties of complex, multicellular circadian systems. *Sci. Adv.* **2023**, *9*, eadh1308, doi: https://doi.org/10.1126/sciadv.adh1308.
